# Disease-associated *KIF3A* variants alter gene methylation and expression impacting skin barrier and atopic dermatitis risk

**DOI:** 10.1038/s41467-020-17895-x

**Published:** 2020-08-14

**Authors:** Mariana L. Stevens, Zhonghua Zhang, Elisabet Johansson, Samriddha Ray, Amrita Jagpal, Brandy P. Ruff, Arjun Kothari, Hua He, Lisa J. Martin, Hong Ji, Kathryn Wikenheiser-Brokamp, Matthew T. Weirauch, Dorothy M. Supp, Jocelyn M. Biagini Myers, Gurjit K. Khurana Hershey

**Affiliations:** 1 Division of Asthma Research, Cincinnati Children’s Hospital Medical Center, Cincinnati, OH 45229 USA; 2 Division of Human Genetics, Cincinnati Children’s Hospital Medical Center, Cincinnati, OH 45229 USA; 3 Department of Pediatrics, University of Cincinnati College of Medicine, Cincinnati, OH 45229 USA; 4 Division of Pathology and Laboratory Medicine, Cincinnati Children’s Hospital Medical Center, Cincinnati, OH 45229 USA; 5 Division of Pulmonary Biology, Cincinnati Children’s Hospital Medical Center, Cincinnati, OH 45229 USA; 6 Division of Center for Autoimmune Genomics and Etiology, , Cincinnati Children’s Hospital Medical Center, Cincinnati, OH 45229 USA; 7 Division of Biomedical Informatics, Cincinnati Children’s Hospital Medical Center, Cincinnati, OH 45229 USA; 8 Division of Developmental Biology, Cincinnati Children’s Hospital Medical Center, Cincinnati, OH 45229 USA; 9 Research Department, Shriners Hospitals for Children, Cincinnati, OH 45229 USA; 10 Department of Surgery, University of Cincinnati College of Medicine, Cincinnati, OH 45221 USA

**Keywords:** Gene expression, Genetic markers, DNA methylation, Skin diseases

## Abstract

Single nucleotide polymorphisms (SNPs) in the gene encoding kinesin family member 3A, *KIF3A*, have been associated with atopic dermatitis (AD), a chronic inflammatory skin disorder. We find that *KIF3A* SNP rs11740584 and rs2299007 risk alleles create cytosine-phosphate-guanine sites, which are highly methylated and result in lower *KIF3A* expression, and this methylation is associated with increased transepidermal water loss (TEWL) in risk allele carriers. *Kif3a*^*K14*∆*/*∆^ mice have increased TEWL, disrupted junctional proteins, and increased susceptibility to develop AD. Thus, *KIF3A* is required for skin barrier homeostasis whereby decreased *KIF3A* skin expression causes disrupted skin barrier function and promotes development of AD.

## Abstract (web summary)

Genetic variants in *KIF3A* are associated with atopic dermatitis (AD). Here, the authors identify two AD-risk alleles that show high methylation resulting in lower *KIF3A* expression. Mice with epidermis-specific loss of *Kif3a* show disrupted skin barrier homeostasis and increased AD susceptibility.

## Introduction

Atopic dermatitis (AD) is the most common chronic inflammatory skin disease, affecting up to 20% of children^1^. Although up to 70% of cases spontaneously remit before adolescence, AD often precedes the development of additional allergic co-morbidities later in life, such as asthma and allergic rhinitis, termed the atopic march^1^. Genetic variation in the cilia structural gene kinesin family number 3A (*KIF3A*) has been associated with AD, asthma, and the atopic march by numerous studies^2–8^. *KIF3A* encodes a subunit of the cilia component kinesin-2 and is required for the formation of both motile and nonmotile primary cilia^9,10^. Although *Kif3a* null mice are embryonic lethal^11^, we and others have shown that lung-specific deletion of *Kif3a* results in barrier dysfunction and unleashes a Th2 allergen response, which contributes to the development of an asthma phenotype^12,13^. While *KIF3A* genetic associations with asthma and AD have been replicated, the mechanism underlying this susceptibility is unknown. Among the *KIF3A* SNPs associated with asthma and AD, we have found that a third of them create novel cytosine-phosphate-guanine (CpG) sites^7^. CpG regions are prone to modification of the cytosine by the addition of a methyl group to the 5′ position and are known to regulate gene transcription^14,15^. In fact, genetic variations have been highly correlated with DNA methylation and CpG sites, linking single nucleotide polymorphisms with epigenetics and gene regulation^16,17^.

In the present study, we integrate human and mouse studies to dissect the mechanistic basis for the risk conferred by 2 common SNPs in *KIF3A* for AD. Herein, we show that rs11740584 and rs2299007 AD-risk alleles create new CpG sites, which are highly methylated in individuals carrying one or two copies of the alternate allele. Both SNPs are expression quantitative trait loci (eQTLs) in numerous tissues and allele-specific PCR confirms lower expression from the alternate (risk) allele compared to the reference allele. Methylation levels at these new CpG sites are associated with increased transepidermal water loss (TEWL). These data suggest that *KIF3A* is required for skin barrier homeostasis and decreased *KIF3A* expression results in skin barrier dysfunction. In order to investigate causality, we characterize *Kif3a*^*K14*∆/∆^ mice. Mice with keratinocyte-selective deletion of *Kif3a* have increased TEWL and skin thickness, upregulation of the basal marker keratin 5, and dysregulation of adherens and tight junction markers, E-cadherin and claudin-1, respectively. Further, these mice have increased susceptibility to develop features of AD following cutaneous allergen exposure. Thus, rs11740584 and rs2299007 alter the transcriptional regulation of *KIF3A*, result in decreased *KIF3A* expression, promote skin barrier dysfunction, and increase susceptibility for AD development. *KIF3A* is positioned in the 5q31 region, which contains the Th2 cytokine cluster including *IL4* and *IL13*. As such, the association of *KIF3A* with allergic outcomes is usually attributed to its proximity to the Th2 gene cluster. Our results highlight the independent role of *KIF3A* as a key mechanistic pathway for allergic disease pathogenesis and provide insights into the transcriptional regulation of *KIF3A*.

## Results

### Disease-associated *KIF3A* SNPs are eQTL

**Fig. 1:**
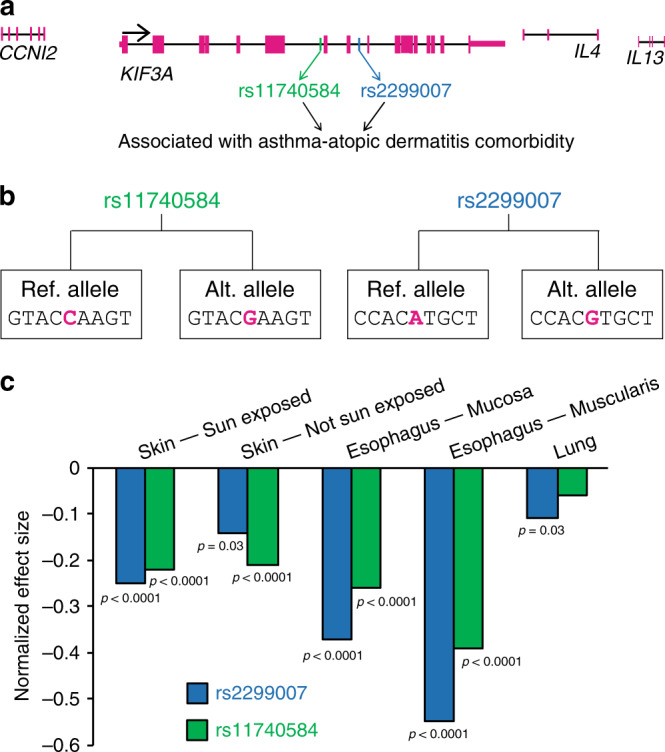
*KIF3A* SNPs rs11740584 and rs2299007 create novel CpG sites and are eQTL. **a** Genomic organization of the human *KIF3A* and neighboring genes on chromosome 5q31 with the location of SNPs rs11740584 and rs2299007 marked. Magenta bars indicate exons. **b** Alternate alleles create new CpG sites in both rs11740584 and rs2299007. **c** Tissue-specific normalized effect size on *KIF3A* expression data for rs11740584 and rs2299007 were retrieved from the GTEx database, analysis release V7 (https://www.gtexportal.org). Each tissue includes biologically independent samples (skin sun exposed *n* = 605, skin not sun exposed *n* = 517, esophagus—mucosa *n* = 497, esophagus—muscularis *n* = 465 and lung *n* = 515). The normalized expression values are based on quantile normalization within each tissue and inverse quantile normalization for each gene across samples. The normalized effect size of the eQTL is defined as the effect of the alternate allele relative to the reference allele in the human genome reference GRCh37/hg19. Source data are provided as a Source Data file.

*KIF3A* SNPs rs11740584 and rs2299007 have been associated with asthma and AD^7^, and both are CpG SNPs, such that the alternate or non-reference allele creates a new CpG site (Fig. 1a, b). Since CpGs undergo epigenetic regulation and impact gene transcription, we utilized Genotype-Tissue Expression Project (GTEx) data^18,19^ to determine whether rs11740584 and rs2299007 are eQTL. The alternate alleles of rs11740584 and rs2299007 had 14–22% decreased *KIF3A* expression in sun-exposed and non-sun-exposed skin compared to the reference allele. In the esophagus, the alternate alleles had 26–55% decreased *KIF3A* expression compared to the reference alleles (Fig. 1c). Moreover, this effect appeared to be tissue-specific, since the SNPs were strong eQTLs in skin and esophagus, but weaker in the lung.

### Alternate alleles generate highly methylated CpG sites

**Fig. 2:**
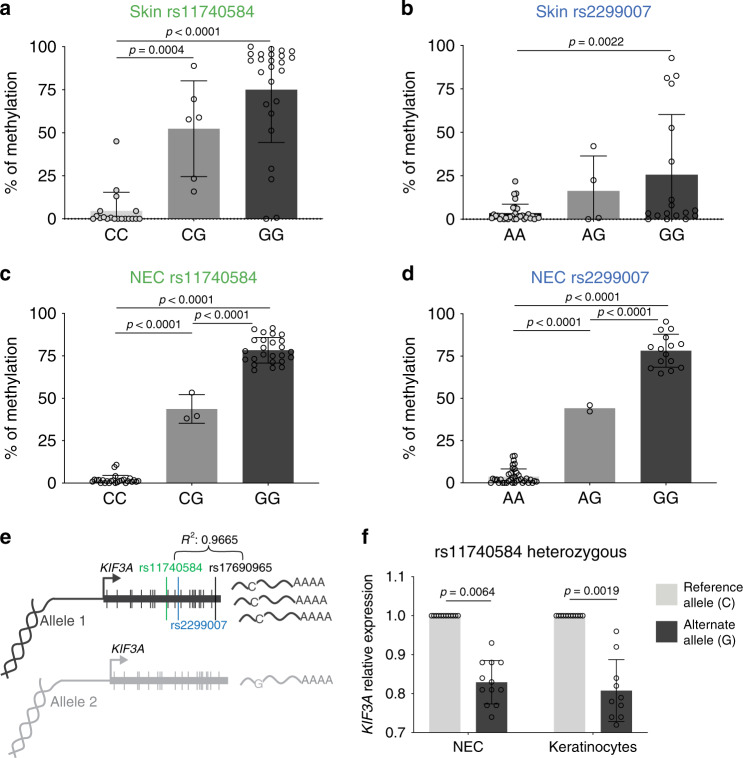
rs11740584 and rs2299007 minor allele creates methylated CpG sites and alter *KIF3A* expression. Skin tape (**a**, **b**), and nasal epithelial cell (**c**, **d**), methylation levels at rs11740584 and rs2299007 CpG sites in homozygous and heterozygous alternate allele carriers. A one-way ANOVA was conducted to compare effects of genotype on methylation levels. Each panel includes *n* = 54 biologically independent samples examined over two independent experiments. Data are presented as mean values ± SD. **e** Experimental design of allele-specific gene expression illustrating the location of the SNPs, their linkage, and the *KIF3A* gene. **f** Fold-change in expression in nasal epithelial and primary keratinocyte cells are provided relative to the C allele. Data are presented as mean values ± SD of three individuals in technical quadruplicates examined over two independent experiments per tissue type. Linear mixed models were performed to access the expression difference between risk allele and reference allele. *P* values of two-sided testing were reported for all the tests. NEC = nasal epithelial cells. Source data are provided as a Source Data file.

In order to investigate the mechanistic basis by which rs11740584 and rs2299007 contribute to AD, we recruited individuals carrying 1 or 2 alternate alleles for both SNPs, as well as age- and sex-matched controls who had 2 copies of the reference allele at both SNPs. The study population included 56 individuals between ages 7 and 26 of both white and non-white races (Table 1). Since rs11740584 and rs2299007 correlated with decreased expression of *KIF3A* (Fig. 1c) and were predicted to create new CpG sites, we hypothesized that the risk alleles for these SNPs may regulate *KIF3A* expression by altering gene methylation. Given *KIF3A* association with AD and asthma^7,8^, we assessed DNA methylation in skin from adhesive tape strips and in nasal epithelial cells (NEC), from human subjects heterozygous or homozygous for the alternate allele. Individuals with 2 reference alleles for rs11740584 or rs2299007 had either very low or negligible methylation at these loci in both skin and NEC. In contrast, individuals with 1 copy of the alternate allele for rs11740584 had an average of 52 and 43% methylation, while rs2299007 heterozygotes had an average of 16 and 44% methylation in skin and NEC, respectively. Individuals with 2 copies of the alternate allele had a range of 25–78% methylation at the newly-created CpG sites (Fig. 2a–d). Thus, the alternate alleles for both rs11740584 and rs2299007 created novel CpG sites and these sites were highly methylated in vivo. Our results also indicate that the methylation levels of rs11740584 and rs2299007 are dependent on the number of alternate alleles, since heterozygotes had an average of half of the methylation level observed in homozygotes at each of the novel CpG sites (Fig. 2a–d). Notably, rs11740584 and rs2299007 genotypes did not result in altered methylation levels at neighboring CpG sites (Supplementary Fig. 1a–c). These data strongly suggest that these specific SNPs might modify *KIF3A* expression by changing gene methylation at the newly-created CpG sites and, thus, transcriptional regulation.

**Table 1:** Clinical and demographic characteristics of individuals carrying the reference vs. alternate alleles of *KIF3A* SNPs rs11740584 and rs2299007.

Clinical variables	rs11740584 Reference^a^ (C)	rs11740584 Alternate^b^ (G)	rs2299007 Reference^a^ (A)	rs2299007 Alternate^b^ (G)
*N*	27	29	38	18
Black	4 (15%)	10 (34%)	12 (32%)	2 (11%)
Male	18 (67%)	19 (66%)	24 (63%)	13 (72%)
Age	15.7 ± 4.2	14.7 ± 3.4	15.3 ± 3.7	15.1 ± 4.1
History of AD	13 (48%)	15 (52%)	18 (47%)	10 (56%)
TEWL^c^	1.03 (0.87–1.54)	1.09 (0.85–1.42)	1.02 (0.86–1.44)	1.14 (0.80–1.56)

^a^Reference allele carriers were homozygous for the reference alleles.

^b^Alternate allele carriers were either heterozygous or homozygous for the alternate alleles.

^c^TEWL measurements were corrected for day-to-day variation by normalization to the same control subject whose TEWL was measured within 30 min of every subject.

### rs11740584 alternate allele decreases *KIF3A* expression

We next utilized allele-specific qPCR to determine whether rs11740584 directly impacts *KIF3A* expression. Since rs11740584 and rs2299007 are intronic and, therefore, not transcribed in messenger RNA, we identified a genetic variant (rs17690965) located within a *KIF3A* exon, which is in strong LD with rs11740584 (*r*^2^ = 0.96) (Fig. 2e). With allelic qPCR, we observed a 17% decrease in *KIF3A* expression for the alternate allele G relative to the reference allele C in NEC derived from three heterozygote individuals (*p* = 0.0064, linear mixed models) (Fig. 2f). We also assessed allele-specific expression in primary human keratinocytes derived from 3 donors heterozygous for rs11740584 and found 19% less expression from the risk allele (*p* = 0.0019, linear mixed models) (Fig. 2f). Thus, the rs11740584 alternate allele results in lower *KIF3A* gene expression compared to the reference allele in primary keratinocytes and upper respiratory NEC. These data are consistent with the GTEx data where the alternate alleles of rs11740584 and rs2299007 were associated with 14–22% decreased expression (Fig. 1c). No coding genetic variant in strong LD was identified for rs2299007.

### In silico analysis of rs11740584 and rs2299007

Since rs11740584 and rs2299007 were associated with increased CpG methylation and decreased expression, we conducted an in silico analysis in order to identify particular transcription factors (TFs) whose binding sites might be altered by the rs11740584 and rs2299007 genetic variants using the CisBP web^20^ and the findings are summarized in Supplementary Table 1. Strikingly, the non-reference (G) allele creates a perfect (consensus) E-box binding site (CACATG changed to CACGTG). Further, ChIP-seq peaks for the MYC E-box binding protein are present in BJ cells (foreskin fibroblast cell line), indicating that MYC is capable of binding this locus in a cellular context^21^. Basic helix-loop-helix (bHLH) family TFs such as MYC can bind the CACGTG E-box when the CpG is methylated^22^. Neither SNP has histone marks in relevant (skin or lung) cell types. The non-reference (risk) allele for rs11740584 creates a binding site for the transcription factor ZSCAN26 (Supplementary Table 1).

### Methylation levels are associated with increased TEWL

We observed that presence of *KIF3A* SNPs rs11740584 and rs2299007 created highly methylated CpG sites and resulted in the downregulation of *KIF3A* expression by about 17–19%. Since these SNPs were strongly associated with AD, we hypothesized that these epigenetic changes in *KIF3A* might be correlated with disrupted skin barrier function in individuals carrying the alternate alleles. To address this, we directly compared skin TEWL and rs11740584 and rs2299007 methylation levels. Methylation levels in the skin of the novel CpG sites created by rs11740584 and rs2299007 were significantly associated with skin barrier dysfunction as assessed by TEWL values (*p* = 0.01 and 0.0004, respectively, generalized linear models) in human carriers of the risk alleles (Table 2).

**Table 2:** *KIF3A* SNPs rs11740584 and rs2299007 methylation levels are associated with higher transepidermal water loss (TEWL).

	*N*	TEWL Estimate	TEWL S.E.	TEWL *P* value
rs11740584 skin methylation	37	0.008	0.003	0.01
rs2299007 skin methylation	47	0.010	0.003	0.0004

*N*: sample size, S.E.: standard error.

The *P* values were adjusted for race, age and sex. Two-sided generalized linear model with Gaussian distribution and log link function were fitted for the analysis.

### *Kif3a* is required for skin barrier homeostasis and function

**Fig. 3:**
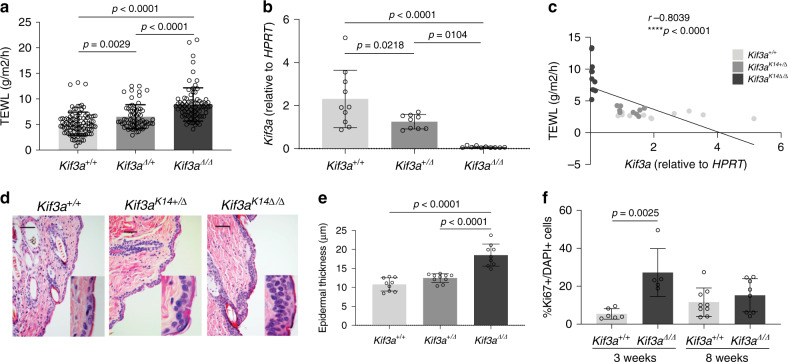
*Kif3a*^*K14*∆/∆^ mice develop disrupted skin barrier. **a** Trans-epidermal water loss (TEWL) was measured in *Kif3a*^+/+^, *Kif3a*^∆/+^ and *Kif3a*^*K14*∆/∆^ mice, age 6–10 weeks (*n* = 284 biologically independent animals examined over three independent experiments). **b** RNA isolated from *Kif3a*^+/+^, *Kif3a*^∆/+^, and *Kif3a*^*K14*∆/∆^ epidermis was assessed for mRNA levels of *Kif3a*. Values are expressed as fold change over *Kif3a*^+/+^ and relative to *Hprt* (*n* = 29 biologically independent animals examined over three independent experiments). A one-way ANOVA was conducted to compare the effects of genotype on TEWL and gene expression. **c** Linear correlation between TEWL and *Kif3a* expression in *Kif3a*^+/+^, *Kif3a*^∆/+^, and *Kif3a*^*K14*∆/∆^ mice (*n* = 30 biologically independent animals examined over three independent experiments). Spearman correlation was conducted to compare TEWL and *Kif3a* expression. **d** Representative H&E staining and **e** epidermal thickness of *Kif3a*^+/+^, *Kif3a*^∆/+^, and *Kif3a*^*K14*∆/∆^ mice at 8 weeks of age (*n* = 28 biologically independent animals examined over three independent experiments). Scale bar: 100 μm. A one-way ANOVA was conducted to compare effects of genotype on epidermal thickness. **f** Ki67 quantification on epidermis sections from 3 and 8 week old *Kif3a*^+/+^ and *Kif3a*^*K14*∆/∆^ mice (*n* = 11 and *n* = 17 biologically independent animals examined over two independent experiments, respectively). *T* test was conducted to compare effects of genotype on Ki67 expression. *P* values of two-sided testing were reported for all the tests. Data in all panels are presented as mean values ± SD. Source data are provided as a Source Data file.

In order to directly prove that reduced *Kif3a* expression causes skin barrier dysfunction, we generated *Kif3a*^*K14*∆/∆^ mice with epidermis-specific deletion of *Kif3a*. *Kif3a*^*K14*∆/∆^ mice had higher TEWL compared to *Kif3a*^*+/+*^ control mice (*p* < 0.0001, one-way ANOVA) (Fig. 3a) demonstrating skin barrier disruption. Notably, intermediate TEWL measurements were observed in *Kif3a*^*K14*∆/+^ mice and were significantly elevated compared to control mice, supporting a gene dosage effect (*p* < 0.01, one-way ANOVA). In fact, we found that skin barrier integrity, assessed by TEWL, was inversely correlated to skin *Kif3a* mRNA levels (*p* < 0.0001, one-way ANOVA) (Fig. 3b, c). Histological examination of skin sections showed intermittent regions of increased thickness in *Kif3a*^*K14*∆/∆^ adult mice relative to *Kif3a*^+/+^ littermates. The typically very thin epidermis in haired areas was increased from the normal 2–3 cell layers to up to 6 cells in thickness. Parakeratosis, hyperkeratosis, and hypergranulosis were focally present in *Kif3a*^*K14*∆/∆^ mice with these findings being rare in *Kif3a*^+/+^ mice (Fig. 3d, e). Epithelial hyperplasia was uniformly present in *Kif3a*^*K14*∆/∆^ mice analyzed at 3 weeks, 5 weeks, and 8 weeks of age and was not seen in age matched *Kif3a*^+/+^ mice (Supplementary Fig. 2a, b). Consistent with what we observed with TEWL, the *Kif3a*^*K14*∆/+^ mice had intermediary epidermal thickness between the *Kif3a*^*K14*∆/∆^ and *Kif3a*^+/+^ mice (Fig. 3e). To address whether epidermal thickness was a result of increased cell proliferation, we carried out immunostaining with Ki67. In 3 week old mice, there was a significant increase in the percentage of Ki67 positive cells in the interfollicular epidermis of *Kif3a*^*K14*∆/∆^ compared to *Kif3a*^+/+^ animals (Fig. 3f; *p* = 0,0025, *t* test). This difference was no longer evident at 8 weeks of age (Fig. 3f) suggesting that the increased epidermal thickness observed in 8-week old animals might be a result of the earlier cell proliferation.

### *Kif3a*-deficient skin has abnormal epidermal cell adhesion

**Fig. 4:**
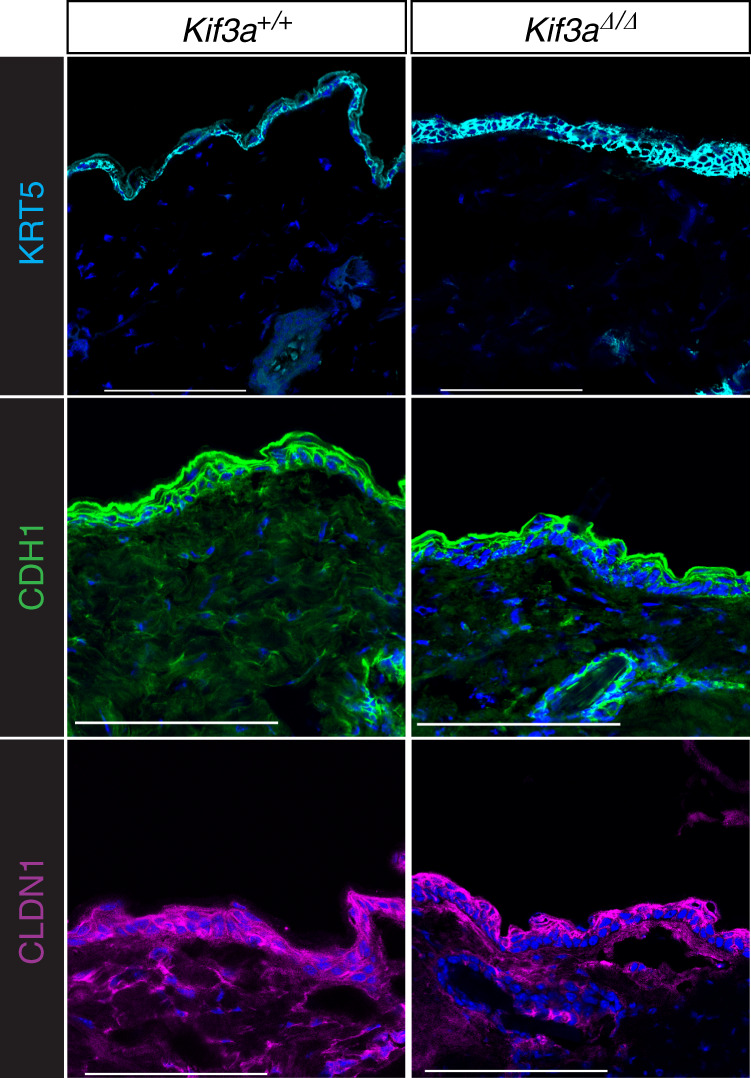
*Kif3a*^*K14∆/∆*^ mice have disrupted adherens junction and tight junction markers. Epidermis sections from 8 to 10 week old *Kif3a*^*+/+*^ and *Kif3a*^*K14*∆/∆^ mice were stained for basal cell marker keratin 5, adherens junction E-cadherin, and tight junction claudin-1, and imaged by confocal microscopy. DAPI was used as nuclei marker. Data shown here is maximum intensity projection. (*n* = 15 biologically independent animals examined over two independent experiments). Scale bar: 100 μm.

Given the disrupted skin barrier observed in the *Kif3a*^*K14*∆/∆^ mice, we next performed transcriptome analyses of the epidermis to gain insights into the observed phenotype in the mice. RNA-seq was performed on 8 week old *Kif3a*^+/+^ and *Kif3a*^*K14*∆/∆^ epidermis. Analysis of differential gene expression revealed 394 downregulated and 408 upregulated genes in the absence of *Kif3a* (Supplementary Fig. 3a–c). Among the top functional terms represented in the downregulated genes were regulation of defense mechanisms, cell adhesion molecule binding, and response to wounding. Meanwhile, the top functional terms among the upregulated genes were growth factor binding and basolateral membrane (Supplementary Fig. 3d). Epidermal expression of *Irf4*, *Wnt7a*, and *Kng2* were downregulated, confirming the downregulation of defense and wound response genes in the absence of *Kif3a*. Meanwhile markers for response to sterol and growth factor binding *Inhba*, *Ntrk4*, and *Igfals* were upregulated in the absence of *Kif3a* (Supplementary Fig. 3e). Collectively, the RNA-seq data indicate skin morphological changes with increased expression of genes expressed in the basolateral membrane and decreased expression of cell adhesion genes in the absence of *Kif3a*, thus we assessed the distribution of basal cell marker keratin 5, as well as adherens junction and tight junction markers E-cadherin and claudin1 in *Kif3a*^+/+^ and *Kif3a*^*K14*∆/∆^ mice. Confocal imaging of skin sections stained for keratin 5 revealed an expansion of the basal layer from 1 to 2 cells found in *Kif3a*^+/+^ animals, to 5–6 cells in *Kif3a*^*K14*∆/∆^ (Fig. 4). Expression of E-cadherin and claudin1 was drastically diminished in the basal cells of *Kif3a*^*K14*∆/∆^ epidermis compared to the even distribution in wild types (Fig. 4). Overall, these data strongly support a role for *Kif3a* in skin homeostasis, whereby loss of *Kif3a* results in over proliferation of the basal cell layer and altered localization of adherens and tight junction proteins that likely contribute to the observed higher TEWL at baseline.

### *Kif3a*^*K14*∆/∆^ mice are prone to develop an AD-like phenotype

**Fig. 5:**
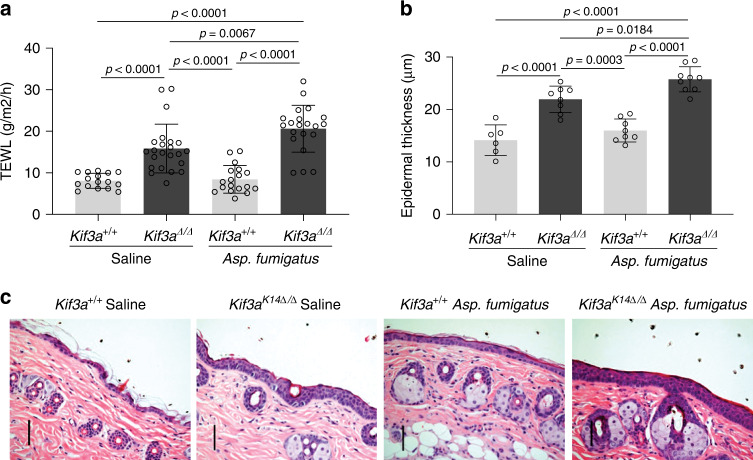
*Kif3a*^*K14*∆/∆^ mice are more prone to develop AD-like phenotype following cutaneous allergen exposure. **a** TEWL measurement at 8 weeks of age in *Kif3a*^+/+^ and *Kif3a*^*K14*∆/∆^ mice sensitized with saline control or *Aspergillus fumigatus* (*n* = 78 biologically independent animals examined over three independent experiments). **b** Epidermal thickness and **c** representative H&E staining of skin samples of *Kif3a*^+/+^ and *Kif3a*^*K14*∆/∆^ mice sensitized with *Aspergillus fumigatus* (*n* = 31 biologically independent animals examined over three independent experiments). Scale bars, 50 μm. A one-way ANOVA was conducted to compare effects of genotype and/or treatment on TEWL and epidermal thickness. *P* values of two-sided testing were reported for all the tests. Data in all panels are presented as mean values ± SD. Source data are provided as a Source Data file.

Since *KIF3A* has been identified as a susceptibility gene for AD^2–8^ and our data revealed that its deficiency causes skin barrier dysfunction, we reasoned that *Kif3a*^*K14*∆/∆^ mice may have increased susceptibility to develop AD. *Kif3a*^*K14*∆/∆^ mice skin appeared normal without lesions or erythema by H&E staining (Fig. 3d) and no baseline inflammation was observed in the skin by *IL-4* and *IL-13* qPCR, and CD3 staining (Supplementary Fig. 4). Therefore, we used a cutaneous allergen exposure of *Aspergillus fumigatus*, to model AD. In this model, cutaneous applications of *Asp. fumigatus* result in increased TEWL, allergic sensitization, skin erythema, skin thickness, and Th2 responses, all landmarks of AD, without the need for prior mechanical tape stripping or skin perturbation with shaving creams needed in other models^23–25^. Upon one week of skin patching, *Kif3a*^*K14*∆/∆^ developed increased TEWL after cutaneous exposure to *Aspergillus fumigatus* allergen compared to *Kif3a*^+/+^ mice (Fig. 5a). Epidermal thickness was also significantly increased in *Kif3a*^*K14*∆/∆^ mice compared to *Kif3a*^+/+^ mice upon cutaneous allergen exposure (Fig. 5b, c). Thus, *Kif3a*^*K14*∆/∆^ mice have a disrupted skin barrier and, while they do not have AD at baseline, they are more susceptible to develop AD-like features upon cutaneous allergen exposure than their wild type counterparts.

## Discussion

**Fig. 6:**
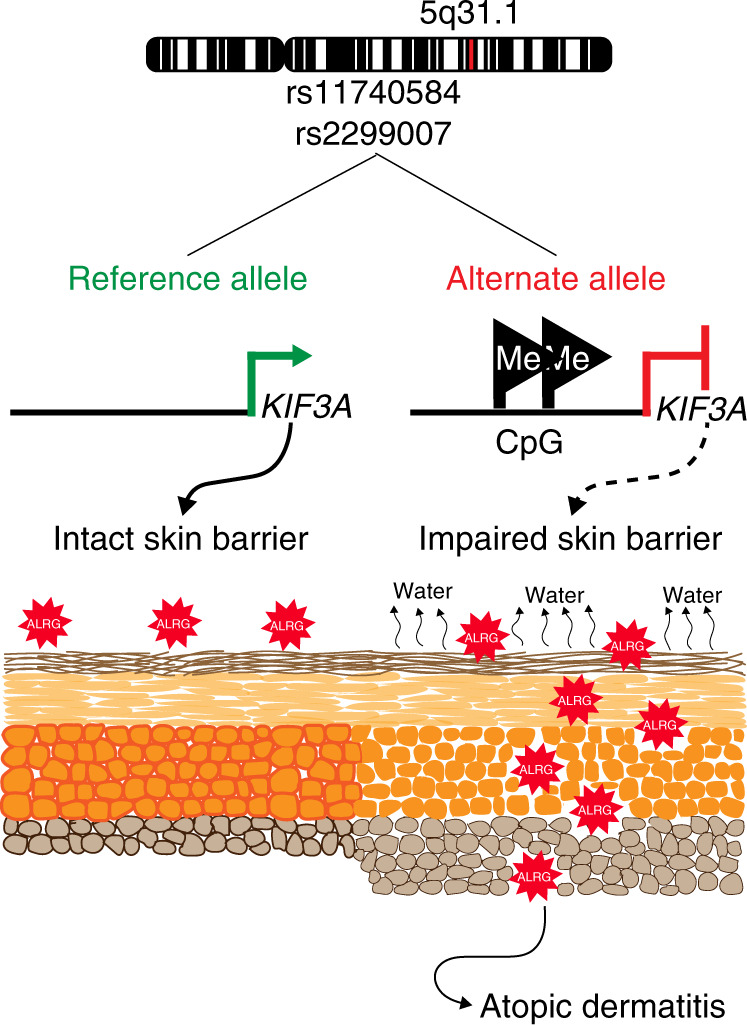
Mechanistic underpinnings of the contribution of *KIF3A* SNPs rs11740584 and rs2299007 to skin barrier dysfunction. Allergic disease-associated *KIF3A* SNPs create new CpG sites, which are highly methylated in individuals carrying the alternate allele leading to decreased *KIF3A* expression. Decreased *KIF3A* results in increased TEWL due to defective cell-cell adhesion, and increased susceptibility to the development of AD. ALRG = allergen.

Our data provide a mechanistic basis for the AD disease susceptibility conferred by *KIF3A* SNPs rs11740584 and rs2299007 whereby the alternate alleles generate new CpG sites resulting in increased methylation and decreased expression of *KIF3A*. Skin methylation of the novel CpG sites created by rs11740584 and rs2299007 was associated with increased TEWL in individuals carrying the risk alleles. To prove causality, we characterized mice with tissue-specific deficiency of *Kif3a* in keratinocytes. *Kif3a*^*K14*∆/∆^ mice had skin barrier dysfunction at baseline as evidenced by increased TEWL and epidermal thickness and disrupted basal cell, adherens and tight junctional protein expression. Finally, the skin barrier dysfunction observed in *Kif3a*^*K14*∆/∆^ mice is disease relevant as these mice had increased susceptibility to develop AD features upon cutaneous allergen exposure. Thus, *KIF3A* deficiency causes skin barrier dysfunction and contributes to AD susceptibility (Fig. 6).

Previous work by our group and others has shown that *KIF3A* is a susceptibility gene for AD and asthma^2–8^, however, until now, the mechanistic basis for this association has been unclear. *KIF3A* rs11740584 and rs2299007 alternate alleles are predicted to create new CpG sites. Although CpG regions are susceptible to DNA methylation, they do not necessarily receive the methyl group^14,15,26^, thus the generation of a CpG does not mean that they are actually methylated in vivo. However, we found that alternate allele carriers for rs11740584 and rs2299007 had 25–78% methylation at each of these sites (vs. 2–5% in reference allele carriers). The high methylation level at these SNP loci in human alternate allele carriers suggests that these SNPs are important in the transcriptional regulation of *KIF3A*. Indeed, both rs11740584 and rs2299007 are eQTLs for *KIF3A* in different tissues of interest and allele-specific PCR revealed that rs11740584 alternate allele results in up to 19% less *KIF3A* expression. The non-reference (G) risk allele for rs2299007 creates a CACGTG E-box. Basic helix-loop-helix (bHLH) family TFs, such as MYC can bind the CACGTG E-box when the CpG is methylated^22^. So, the prediction would be that the rs2299007 non-reference risk allele creates a binding site for MYC, and MYC binds this site more strongly. Indeed, rs2299007 is located inside of a MYC ChIP-seq peak in fibroblasts^21^. Collectively, these findings add a layer to what is currently known regarding *KIF3A* transcriptional regulation and further highlight the relevance of our findings. Chromatin modifications, and RNA polymerase II loading have all been shown to be modified by SNPs^27,28^, and could be the link between these variants and transcriptional regulation. Our data demonstrate that the new sites are methylated in alternate allele carriers, while they are not methylated in reference allele carriers. Thus, *KIF3A* rs11740584 and rs2299007 genotypes predict the methylation status at the SNP site and support a role in *KIF3A* gene expression regulation. Although we showed strong evidence for this correlation, we did not directly test that methylation is associated with expression independent of genotype. It remains to be elucidated how *KIF3A* rs11740584 and rs2299007 CpG methylation alters *KIF3A* expression and what the transcriptional regulatory events are that mediate this process.

While our data demonstrate that *KIF3A* can contribute to AD development, it is important to note that *KIF3A* or any single factor is highly unlikely to be solely responsible for AD development. *KIF3A* is a component of the primary cilia, which are sensory structures that transduce extracellular signals to the cell interior^9,10^, and in *Kif3a* absence, this microtubule-based structure is not formed properly^29^. Because *Kif3a* is important for the primary cilia structure, signaling pathways like Shh, Wnt, and Notch, that rely on this sensory organ^30–32^, might be related to the phenotype described in the present study. Indeed, mice deficient in Notch signaling develop dry skin, barrier abnormalities, and increased TEWL just like reported in the current study^33,34^. Therefore, the role of *Kif3a* in the primary cilia may be to connect different signaling pathways suggesting a complex and intricate regulatory network, that remains to be investigated.

Since *KIF3A* methylation at the newly generated rs11740584 and rs2299007 CpG sites was highly correlated with skin barrier dysfunction, specifically increased TEWL, *KIF3A* genotype and/or methylation at these sites may be clinically useful biomarkers of AD development in pregnancy or early life. This is highly relevant because AD is a significant risk factor for food allergy development, as it has been widely described and systematically reviewed recently^35^. Food allergy prevalence is on the rise, and the development and implementation of strategies for food allergy prevention will require tools to accurately predict which children will develop AD. Indeed, the cytokine gene cluster on chromosome 5q31.1, where *KIF3A* is located, has been associated with food allergies in a GWAS (genome-wide association study) study^36^.

Our findings provide insights into the transcriptional regulation of *KIF3A*. The linkage of chromosomal region 5q31 and the linkage of this locus to allergic disease has been largely attributed to the Th2 cytokine gene cluster^8,37,38^. Our results in mice highlight the independent role of *KIF3A* as a key mechanistic pathway for allergic disease susceptibility and provide insights into the transcriptional regulation of *KIF3A* as wells as a potential new target for the prevention and treatment of AD.

## Methods

### Subjects

The GCPCR- *KIF3A* cohort included a total of 56 participants, 45 children aged 7–17 years old and 11 adults aged 18–26 years old from the greater Cincinnati, Ohio metro area who were initially enrolled in either the Greater Cincinnati Pediatric Clinic Repository (GCPCR), Genomic Control Cohort (GCC), Cincinnati Childhood Allergy and Air Pollution Study (CCAAPS), or the Cincinnati Center for Eosinophilic Disorders (CCED) cohorts, which have been extensively described previously^5,39,40^. Based on genotyping information available from these cohorts, we recruited individuals based on genotype for *KIF3A* SNPs rs11740584 and/or rs2299007. Cases carried at least alternative allele for one of these SNPs, controls did not carry alternatives alleles for either SNP. The cases and controls were matched on age, sex, and allergic disease status. The subjects or their legally authorized representatives (LARs) completed our validated New Visit Questionnaire (NVQ)^39^, which collects information regarding AD, allergic rhinitis, food allergy, asthma and allergy symptoms, medical history, family history of allergic disease, medications, diet, home characteristics, and environmental exposures. Subjects also completed our Demographic Data Questionnaire (DDQ)^39^, which captures age, sex, race, and ethnicity of the participant and participant’s parents and socioeconomic status indicators.

### Study approval

GCPCR-*KIF3A* subjects, or their LAR, provided informed consent or parental permission/assent, respectively, prior to inclusion in the study. The study was approved by the Cincinnati Children’s Hospital Medical Center Institutional Review Board under protocol number 2010-0438. Primary human keratinocytes were isolated from de-identified human discard skin isolates from plastic surgeries at University of Cincinnati, Cincinnati Children’s Hospital Medical Center and Shriners Hospital. The study was approved by the University of Cincinnati Institutional Review Board under protocol number 2013-4582 and determined as “non-human subjects research” by the University of Cincinnati Institutional Review Board. Mice were maintained and handled according to the Guide for the Care and Use of Laboratory Animals (Institute of Laboratory Animal Resources, National Research Council). All animal procedures were approved by the Institutional Animal Care and Use Committee (Cincinnati Children’s Hospital Medical Center).

### Human transepidermal water loss (TEWL)

Water loss was measured using the SkinLab Combo and based on Nilsson’s Vapor Pressure Gradient method. Measurements were made according to published guidelines^41^. The temperature and the relative humidity of the rooms where measurements were made varied between approximately 20 and 27 °C and 10% and 60% relative humidity. Median values of temperature and relative humidity did not differ significantly either between cases and controls (Supplementary Table 2, Wilcoxon test). Subjects had not used any topical ointment, cream, lotion or moisturizer for at least 12 h prior to the measurement, by their report. TEWL measurements were taken after an acclimatization period of at least 15 min. Measurements were recorded as g/m^2^/h after the rate of TEWL had stabilized, approximately after 60 s and when the standard deviation became 0.2 or less. The volar forearm sites were assessed for TEWL. The same coordinator measured TEWL on all study subjects. TEWL measurements were corrected for day-to-day variation by normalization to one control subject whose TEWL was measured within 30 min of every subject.

### Human skin sampling and DNA extraction

Skin cells were collected with adhesive SmartSolve Strips pre-cut into eleven 1 × 1 inch strips. The tape strips were placed adhesive side down on (volar forearm) and massaged for 20 s. The location of the first tape was outlined on the skin and subsequent tapes were applied at the same location. Tapes are collected in BL + TG buffer (4 M guanidine thiocyanate, 0.01 M Tris (pH7.5) and 2% 1-thioglycerol), flash frozen and stored at −80 °C. Genomic DNA was isolated from tape strips using the Promega Wizard DNA isolation kit (Madison, WI).

### Human nasal epithelial cells collection and DNA/RNA extractions

NEC sampling was performed by using a CytoSoft Brush (Medical Packaging Corp) and the sample was immediately placed on ice in 10%ß-ME in RLT buffer (Qiagen AllPrep DNA/RNA Micro Kit). Within 30 min of collection, the sample was flash frozen with dry ice and stored at −80 °C. DNA and RNA were extracted using the AllPrep DNA/RNA Micro Kit (Qiagen).

### Bisulfite pyrosequencing

For DNAm measurement, 300 ng genomic DNA was subjected to sodium bisulfite treatment and purified using the EZ DNA methylation-Gold Kit (Zymo Research) according to the manufacturer’s specifications. Bisulfite DNA was amplified with ZymoTaq PreMix (Zymo Research) with the primer sets listed in the Supplementary Table 3. Pyrosequencing was carried out using Pyro Gold reagents with a PyroMark vacuum prep workstation and a PyroMark Q96 MD instrument (Qiagen) following the manufacturer’s instructions. The generated pyrograms were automatically analyzed using Pyro Q-CpG methylation analysis software (Qiagen, Version 1.0.11). 100% methylation control (SssI-treated human genomic DNA) and 0% methylation control (human genomic DNA amplified by GenomePlex® Complete WGA kit (Sigma) were used in validating all assays.

### Primary human keratinocytes strains

Primary human keratinocytes were isolated from de-identified human skin discarded from breast reductions performed in healthy females. Cells were isolated from human epidermis, and were confirmed to be keratinocytes based on morphology and growth characteristics in selective culture media.

### Allelic qPCR

The SNPs rs11740584 and rs2299007 are intronic and, therefore, not transcribed in messenger RNA, so we identified a genetic variant (rs17690965) in strong LD with rs11740584 (*r*^2^ = 0.96) located within a *KIF3A* exon. No genetic variant in strong LD was found for rs2299007 to perform this analysis. NEC and primary human keratinocytes mRNA was used to perform reverse transcriptase reaction with the SuperScript IV VILO Master Mix (Invitrogen). Allele-specific qPCR was performed with Taqman genotyping primers (ThermoFisher) for rs17690965 on keratinocytes and NEC cDNA and genomic DNA of three heterozygote individuals each who carried one alternate and one reference allele. Primer/probe is listed in Supplementary Table 3. Fold change of expression was calculated with 2^−ΔΔCT^ values, where cDNA was normalized to gDNA.

### *KIF3A* associated SNPs in silico transcription factor binding site analysis

We sought to identify particular transcription factors (TFs) whose binding sites might be altered by the rs11740584 and rs2299007 genetic variants using the CisBP web^20^.

### Generation of *Kif3a*^*K14*∆/∆^ mice with targeted deletion of *Kif3a* in the skin

Skin-specific conditional *Kif3a* knockout mice were generated by breeding K14-Cre;R26R-eYFPflox-stop-flox mice^42^ to *Kif3a* flox/flox mice^43^. In the presence of K14-driven Cre-recombinase, flox-flanked exon 2 of the *Kif3a* gene was deleted in epidermal cells introducing a frameshift in the coding region resulting in premature termination of *Kif3a* translation. *Kif3a*^*K14*∆/∆^ mice were maintained on an alternating 12-h light cycle with food and water ad libitum, the temperature was kept between 70 and 74 F and humidity between 30 and 70%, and otherwise maintained and handled according to the Guide for the Care and Use of Laboratory Animals (Institute of Laboratory Animal Resources, National Research Council).

### Mice measurement of TEWL

TEWL was measured by using DermaLab’s instrument (Cortex Technology). The temperature and relative humidity in the rooms are held constant. Mice were shaved one day before the measurement. TEWL was assessed by placing the probe against the shaved skin surface in the center of the back area. Measurements were recorded in g/m^2^/h. TEWL numbers were read twice and the average was recorded.

### Mice skin RNA isolation and quantitative real time PCR

Total RNA was isolated from homogenized mouse skin using RNeasy Microarray Tissue Mini Kit (Qiagen) according to manufactures’ instructions. Skin RNA was used to perform reverse transcriptase reaction with the SuperScript IV VILO Master Mix (Invitrogen). Taqman primer/probes for *HPRT, Kif3a, IL4, IL13, Irf4, Wnt7a, Kng2, Inhba, Ntkr2*, and *Igfals* are listed in Supplementary Table 3. Primers/probes were used following the manufacturer’s instructions. *HPRT* was used as a housekeeping gene to normalize the expression of all genes.

### Mice epidermis RNA isolation

Epidermis from 8 week old *Kif3a*^+/+^ and *Kif3a*^*K14*∆/∆^ animals were isolated with the use of dispase. Total RNA was isolated from homogenized skin using RNeasy Microarray Tissue Mini Kit (Qiagen) according to the manufactures’ instructions.

### RNA-seq

Skin mRNA from six *Kif3a*^+/+^ and six *Kif3a*^*K14*∆/∆^ were submitted to RNA-seq at the Genomics, Epigenomics and Sequencing Core at the University of Cincinnati. The RNA quality was determined by Bioanalyzer (Agilent, Santa Clara, CA). NEBNext Poly(A) mRNA Magnetic Isolation Module (New England BioLabs, Ipswich, MA) was used to isolate polyA RNA. Library was prepared with NEBNext Ultra II Directional RNA Library Prep Kit (New England BioLabs) and sequenced using HiSeq 1000 sequencer (Illumina, San Diego, CA). Each sample had around 25 million single reads at 51 bp long. Quality trimmed reads were mapped to the mm10 genome, quantified using RSEM and mapped with Bowtie 2 using default thresholds^44^. Differential gene expression analysis was carried out with RUVSeq^45^ with log2 fold change (FC) ≥ 1 or ≤ −1, *P* < 0.05 and false discovery rate (FDR) ≤ 5%. Volcano Plots were generated using CSBB’s InteractiveScatterPlot module. GO term enrichment analyses were performed using WebGestalt^46^. Heatmaps were generated using GeneE from Broad Institute (https://software.broadinstitute.org/GENE-E/index.html).

### Mice skin histology

Skin tissues were fixed in 10% formalin immediately after mice were euthanized. Paraffin-embedded tissues were cut into 5 µm sections and stained with H&E for histology and to assess skin thickness. Epidermal thickness was quantified using morphometric software (Metamorph) and an average of 10 random fields (×200) were measured for each sample. T cells were assessed by immunohistochemistry using anti-CD3 (Biolegend). Positive cells were quantified under microscope for 10 to 15 random fields (×200) and results were expressed as cells per field.

### Immunostaining and imaging experiments

Immunostaining experiments were carried out on 7 micron frozen sections of mice epidermis. Air-dried frozen sections were post fixed in 4% paraformalydehyde. Sections were incubated with following primary antibodies: Keratin 5 (905501, Rabbit polyclonal, Biolegend), E-cadherin (180223, Mouse monoclonal IgG1kappa, Life technologies), Claudin 1 (37-4900, Mouse IgG1, Thermofisher Scientific), Ki67 (ab15580, Rabbit polyclonal, Abcam). Primary antibodies were used at 1:200 dilution in 4% NGS block overnight. Sections were incubated with the following secondaries: Alexafluor 488 anti-rabbit (A-11008, Thermofisher Scientific), Alexafluor 488 (A-11029, Thermofisher Scientific), Alexafluor 594 anti-mouse (A-11032, Thermofhisher scientific) and nuclear stain DAPI at 1:200. Confocal imaging was performed using the Nikon TI-E Inverted microscope at the Cincinnati Children’s Confocal Imaging Core. Images were analyzed using the Nikon Imaging Software Elements (Version 4.4) and ImageJ (Version 1.52) was used to quantify immunostaining images as appropriate.

### Mice epicutaneous exposure to *aspergillus fumigatus*

Mice were shaved on their backs a day before the allergen exposure. Sterile 2 × 2 cm gauzes moistened with 200 μg *Aspergillus fumigatus* spore extract (Greer Laboratories) in phosphate-buffered saline was applied to the shaved area. The patch was secured with TegaDerm and a bandaid was placed around the mouse waist. After 6 days the patch was removed, and 24 h later TEWL was measured over the exposed area.

### Statistical analysis

Descriptive statistics were used to characterize the study population. Frequencies (percentage) were reported for categorical variables while geometric means and confidence intervals (CI) were reported for continuous variables as data were log transformed to improve distributional characteristics. Generalized Linear Models with gaussian distribution and log link function were fitted to examine the SNP methylation association with TEWL. The associations between *KIF3A* SNPs genotypes and their methylation were tested using one-way ANOVA. To compare *KIF3A* allelic expression differences, we first standardized the expression data for each person to ensure that the expression data were in the same scale and comparable using the scale function in R. Linear mixed models were performed to access the expression difference between risk allele and reference allele, assuming a positive-definite symmetrical correlation matrix for the two conditions. “Person” was included as a random effect in the (linear mixed) model to account for the correlations among the replicates for each person. Replicate values from 2 different individuals that exceeded a coefficient of variation of 10 were removed. Linear regression was used for the mice analysis to examine *Kif3a* association with TEWL. A one-way ANOVA was conducted to compare effects of genotype on TEWL, gene expression and epidermal thickness (8 week old). T test was conducted to compare effects of genotype on Ki67 expression and epidermal thickness (3 and 5 week old). A nominal *p* value threshold (*p* < 0.05) was applied for significance. *P* values of two-sided testing were reported for all the tests. All analyses were performed in R software, version 3.3.2 (https://www.r-project.org).

### Reporting summary

Further information on research design is available in the Nature Research Reporting Summary linked to this article.

## Supplementary information

Supplementary Information

Peer Review File

Reporting Summary

## Data Availability

Tissue-specific *KIF3A* expression data for rs11740584 and rs2299007 were retrieved from the GTEx database, analysis release V7 (https://www.gtexportal.org). The whole-genome-sequencing data associated with this study are available through GEO accession GSE151706. The source data underlying Figs. 1c, 2a–d, f, 3a–c, e, f, 5a–b, and Supplementary Figs. 1b, c, 2b, 3b, e, 4a, b, d are provided as a Source Data file. All other data supporting the findings of this study are available within the paper and its Supplementary information files.

## Source data

Source Data

## References

[1] Weidinger S, Novak N (2016). Atopic dermatitis. Lancet.

[2] Lepre T (2013). Association of KIF3A, but not OVOL1 and ACTL9, with atopic eczema in Italian patients. Br. J. Dermatol.

[3] Hirota T (2012). Genome-wide association study identifies eight new susceptibility loci for atopic dermatitis in the Japanese population. Nat. Genet..

[4] Kim M (2017). Corrigendum: KIF3A binds to beta-arrestin for suppressing Wnt/beta-catenin signalling independently of primary cilia in lung cancer. Sci. Rep..

[5] Kovacic MB (2011). Identification of KIF3A as a novel candidate gene for childhood asthma using RNA expression and population allelic frequencies differences. PLoS ONE.

[6] Michel S (2010). Unifying candidate gene and GWAS approaches in asthma. PLoS ONE.

[7] Johansson E (2017). KIF3A genetic variation is associated with pediatric asthma in the presence of eczema independent of allergic rhinitis. J. Allergy Clin. Immunol..

[8] Marenholz I (2015). Meta-analysis identifies seven susceptibility loci involved in the atopic march. Nat. Commun..

[9] Hirokawa N (2000). Stirring up development with the heterotrimeric kinesin KIF3. Traffic.

[10] Hirokawa N, Noda Y, Tanaka Y, Niwa S (2009). Kinesin superfamily motor proteins and intracellular transport. Nat. Rev. Mol. Cell Biol..

[11] Marszalek JR, Ruiz-Lozano P, Roberts E, Chien KR, Goldstein LS (1999). Situs inversus and embryonic ciliary morphogenesis defects in mouse mutants lacking the KIF3A subunit of kinesin-II. Proc. Natl Acad. Sci. USA.

[12] Giridhar PV (2016). Airway epithelial KIF3A regulates Th2 responses to aeroallergens. J. Immunol..

[13] Geng G (2018). KIF3A knockdown sensitizes bronchial epithelia to apoptosis and aggravates airway inflammation in asthma. Biomed. Pharmacother..

[14] Feinberg AP (2010). Genome-scale approaches to the epigenetics of common human disease. Virchows Arch..

[15] Zhang X (2017). Nasal DNA methylation differentiates corticosteroid treatment response in pediatric asthma: a pilot study. PLoS ONE.

[16] Zhi D (2013). SNPs located at CpG sites modulate genome-epigenome interaction. Epigenetics.

[17] Shoemaker R, Deng J, Wang W, Zhang K (2010). Allele-specific methylation is prevalent and is contributed by CpG-SNPs in the human genome. Genome Res..

[18] Bahcall OG (2015). Human genetics: GTEx pilot quantifies eQTL variation across tissues and individuals. Nat. Rev. Genet..

[19] Carithers LJ, Moore HM (2015). The Genotype-Tissue Expression (GTEx) Project. Biopreserv. Biobank.

[20] Lambert SA (2019). Similarity regression predicts evolution of transcription factor sequence specificity. Nat. Genet.

[21] Soufi A, Donahue G, Zaret KS (2012). Facilitators and impediments of the pluripotency reprogramming factors’ initial engagement with the genome. Cell.

[22] Yin, Y. et al. Impact of cytosine methylation on DNA binding specificities of human transcription factors. *Science*10.1126/science.aaj2239 (2017). 10.1126/science.aaj2239 PMC8009048 28473536

[23] Akei HS (2006). Epicutaneous aeroallergen exposure induces systemic TH2 immunity that predisposes to allergic nasal responses. J. Allergy Clin. Immunol..

[24] Akei HS, Mishra A, Blanchard C, Rothenberg ME (2005). Epicutaneous antigen exposure primes for experimental eosinophilic esophagitis in mice. Gastroenterology.

[25] Brandt EB, Gibson AM, Bass S, Rydyznski C, Khurana Hershey GK (2013). Exacerbation of allergen-induced eczema in TLR4- and TRIF-deficient mice. J. Immunol..

[26] Smith ZD, Meissner A (2013). DNA methylation: roles in mammalian development. Nat. Rev. Genet..

[27] Lee MP (2012). Allele-specific gene expression and epigenetic modifications and their application to understanding inheritance and cancer. Biochim Biophys. Acta.

[28] Cavalli M (2016). Allele-specific transcription factor binding to common and rare variants associated with disease and gene expression. Hum. Genet..

[29] Kodani A, Salome Sirerol-Piquer M, Seol A, Garcia-Verdugo JM, Reiter JF (2013). Kif3a interacts with Dynactin subunit p150 Glued to organize centriole subdistal appendages. EMBO J..

[30] Croyle MJ (2011). Role of epidermal primary cilia in the homeostasis of skin and hair follicles. Development.

[31] Eggenschwiler JT, Anderson KV (2007). Cilia and developmental signaling. Annu Rev. Cell Dev. Biol..

[32] Ezratty EJ (2011). A role for the primary cilium in Notch signaling and epidermal differentiation during skin development. Cell.

[33] Melnik BC (2015). The potential role of impaired Notch signalling in atopic dermatitis. Acta Derm. Venereol..

[34] Yockey LJ (2013). The absence of a microbiota enhances TSLP expression in mice with defective skin barrier but does not affect the severity of their allergic inflammation. J. Invest. Dermatol.

[35] Tsakok T (2016). Does atopic dermatitis cause food allergy? A systematic review. J. Allergy Clin. Immunol..

[36] Marenholz I (2017). Genome-wide association study identifies the SERPINB gene cluster as a susceptibility locus for food allergy. Nat. Commun..

[37] Myers RA (2014). Genome-wide interaction studies reveal sex-specific asthma risk alleles. Hum. Mol. Genet.

[38] Paternoster L (2011). Meta-analysis of genome-wide association studies identifies three new risk loci for atopic dermatitis. Nat. Genet.

[39] Butsch Kovacic M (2012). The Greater Cincinnati Pediatric Clinic Repository: a novel framework for childhood asthma and allergy research. Pediatr. Allergy Immunol. Pulmonol..

[40] LeMasters GK (2006). High prevalence of aeroallergen sensitization among infants of atopic parents. J. Pediatr..

[41] Pinnagoda J, Tupker RA, Agner T, Serup J (1990). Guidelines for transepidermal water loss (TEWL) measurement. A report from the Standardization Group of the European Society of Contact Dermatitis. Contact Dermat..

[42] McCauley HA (2014). TGFbeta signaling inhibits goblet cell differentiation via SPDEF in conjunctival epithelium. Development.

[43] Lin F (2003). Kidney-specific inactivation of the KIF3A subunit of kinesin-II inhibits renal ciliogenesis and produces polycystic kidney disease. Proc. Natl Acad. Sci. USA.

[44] Li B, Dewey CN (2011). RSEM: accurate transcript quantification from RNA-Seq data with or without a reference genome. BMC Bioinforma..

[45] Risso D, Ngai J, Speed TP, Dudoit S (2014). Normalization of RNA-seq data using factor analysis of control genes or samples. Nat. Biotechnol..

[46] Wang J, Vasaikar S, Shi Z, Greer M, Zhang B (2017). WebGestalt 2017: a more comprehensive, powerful, flexible and interactive gene set enrichment analysis toolkit. Nucleic Acids Res..

