# Supplementary material for: Disease-associated KIF3A variants alter gene methylation and expression impacting skin barrier and atopic dermatitis risk
Source: Nat Commun. 2020 Aug 14;11:4092. doi: 10.1038/s41467-020-17895-x (PMC7427989; doi:10.1038/s41467-020-17895-x)
Supplement: Supplementary file 1 — Supplementary Information [file 41467_2020_17895_MOESM1_ESM.pdf]

Supplementary Information

Disease-associated *KIF3A* variants alter gene methylation and expression impacting skin barrier and atopic dermatitis risk

Stevens et al.

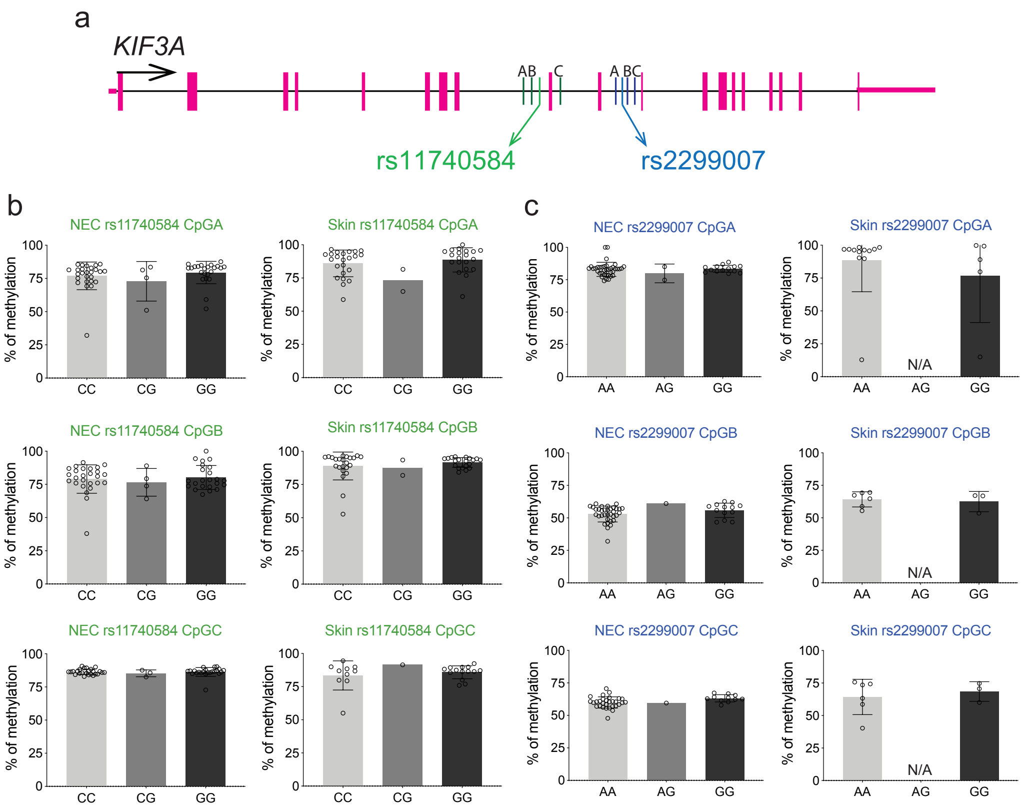

Supplementary Figure 1. Methylation levels of CpGs neighboring *KIF3A* SNPs rs11740584 and rs2299007 are not altered by rs11740584 and rs2299007 genotype. a, Genomic organization of the human *KIF3A* SNPs rs11740584 and rs2299007 and neighboring CpGs. Magenta bars indicate exons. **b-c,** Skin cell (isolated from skin tapes) and nasal epithelial cell methylation levels at rs11740584 and rs2299007 neighboring CpG sites in homozygous and heterozygous alternate allele carriers. Each chart includes biologically independent samples examined over 2 independent experiments (NEC rs11740584 CpGA n=53, CpGB n=53, CpGC n=51; Skin rs11740584 CpGA n=45, CpGB n=45, CpGC n=25; NEC rs2299007 CpGA n=53, CpGB n=48, CpGC n=42; Skin rs2299007 CpGA n=17, CpGB n=9 and CpGC n=9). A one-way ANOVA was conducted to compare effects of genotype on methylation levels. No statistical significance was observed. Data in all panels are presented as mean values +/- SD. NEC = Nasal epithelial cells. Source data are provided as a Source Data file.

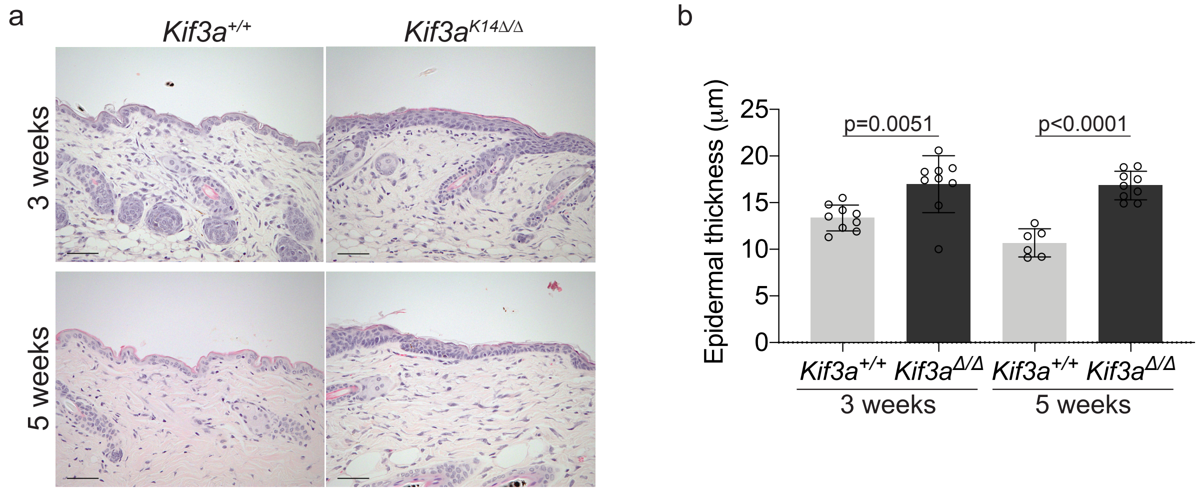

Supplementary Figure 2. *Kif3a*$^{K14\Delta/\Delta}$ mice have increased epidermal thickness. a, Representative H&E staining of *Kif3a*$^{+/+}$ and *Kif3a*$^{K14\Delta/\Delta}$ mice at 3 and 5 weeks of age. **b,** Epidermal thickness quantification of mice at 3 and 8 weeks of age (n=18 and n=15 biologically independent animals examined over 2 independent experiments). Scale bar: 50μm. Two-sided T test was conducted to compare effects of genotype on epidermal thickness. Data is presented as mean values +/- SD. Source data are provided as a Source Data file.

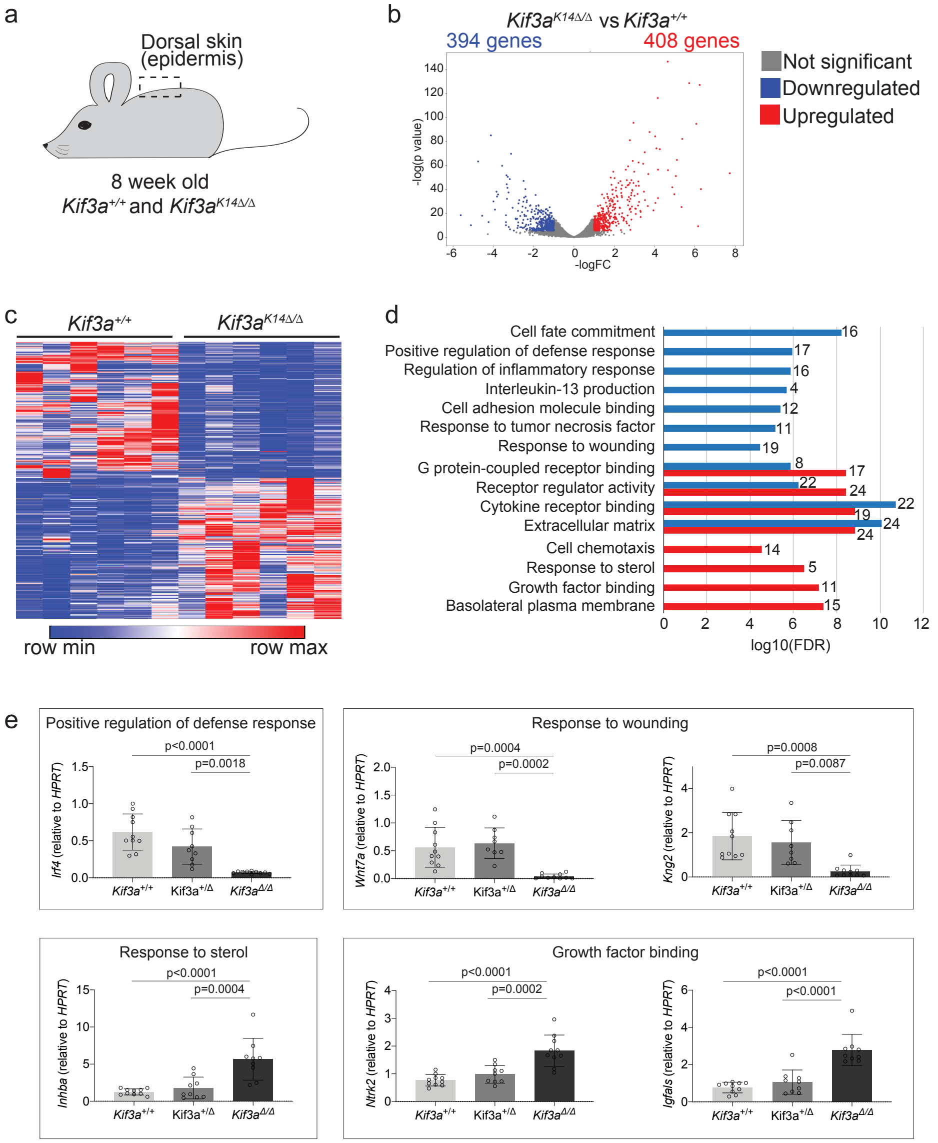

Supplementary Figure 3. Unbiased skin transcriptome analysis of *Kif3a*$^{K14\Delta/\Delta}$ mice. a, Experimental design of the RNA-seq analysis (n=12 biologically independent animals). **b,** Volcano plot of log fold change in expression between *Kif3a*$^{K14\Delta/\Delta}$ versus *Kif3a*$^{+/+}$. Upregulated genes in red and downregulated genes in blue, based on log2 fold change (FC) ≤-1 or ≥1 and false discovery rate (FDR) ≤5%. **c,** Heatmap showing all differentially expressed genes. **d,** GO term enrichment analysis of upregulated (red) and downregulated (blue) genes. **e,** qPCR validation of *Kif3a* regulated genes in *Kif3a*$^{+/+}$, *Kif3a*$^{+/\Delta}$ and *Kif3a*$^{K14\Delta/\Delta}$ 8 week old animals (n=29 biologically

independent animals per panel, examined over 1 experiment). A one-way ANOVA was conducted to compare effects of genotype on gene expression. Data in all panels are presented as mean values +/- SD. Source data are provided as a Source Data file.

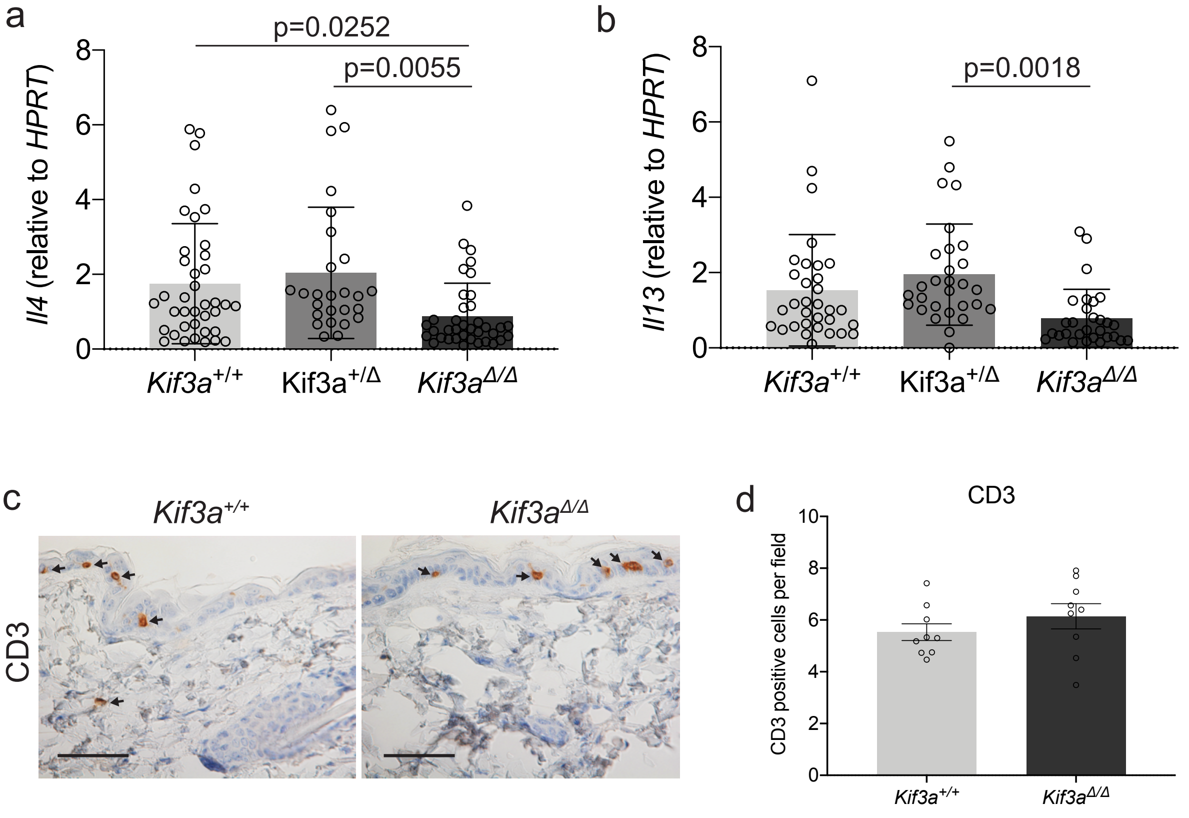

Supplementary Figure 4. *$Kif3a^{K14\Delta/\Delta}$* mice do not have increased inflammation at baseline. **a-b,** RNA isolated from *$Kif3a^{+/+}$*, *$Kif3a^{\Delta/+}$*, and *$Kif3a^{K14\Delta/\Delta}$* epidermis was assessed for mRNA levels of *IL4* and *IL13*. Values are expressed as fold change over *$Kif3a^{+/+}$* and relative to *Hprt* (Il4 n=101 and Il13 n=90 biologically independent animals examined over 4 independent experiments). **c-d,** Skin sections from 8 week old *$Kif3a^{+/+}$* and *$Kif3a^{K14\Delta/\Delta}$* mice were immunostained using anti-CD3 and quantified (n=18 biologically independent animals examined over 1 experiment). Scale bars, 50µm. A one-way ANOVA was conducted to compare effects of genotype on gene expression and CD3 positive cell count. Data in all panels are presented as mean values +/- SD. Source data are provided as a Source Data file.

Supplementary Table 1. Transcription factors whose binding sites are potentially altered by *KIF3A* SNPs. Table shows transcription factors that bind preferentially in the presence of rs11740584 or rs2299007 risk alleles.

rs11740584 risk allele	rs2299007 risk allele
ZSCAN26	ARNT2
	ARNT
	ARNTL
	BHLHE40
	BHLHE41
	CLOCK
	FIGLA
	HES1
	HES2
	HES5
	HES7
	HEY2
	ID1
	ID2
	ID3
	ID4
	MAX
	MITF
	MLX
	MLXIP
	MNT
	MYC
	MYCL
	MYCN
	MYOD1
	SOHLH2
	SREBF1
	SREBF2
	TCF12
	TCF3
	TCF4
	TCFL5
	TFAP4
	TFEB
	TFEC
	TFE3
	USF1/2

Supplementary Table 2. Transepidermal water loss measurements were performed under the same room temperature and humidy conditions during the study. There is no statistical difference in room conditions between clinical visits conducted for *KIF3A* controls and cases.

	rs11740584 median reference x risk allele	rs11740584 p value	rs2299007 median reference x risk allele	rs2299007 p value
Temperature	22.60 x 22.90	0.80	23.00 x 22.40	0.22
Humidity	42.50 x 47.85	0.30	45.45 x 48.20	0.68

Supplementary Table 3. Taqman gene expression primers/probes and PYRO-seq primers used in the study.

ID	Catalog	Sequence
rs11740584 Fwd		5'-AATTTGAGGAAGAAAGTTGATATAGG-3'
rs11740584 Rev		5'-Biotin-AACTTACCCCTTCTTTTTTCCTT-3'
rs11740584 Seq		5'-AGGTATTTTTAAATAGTTAATGAT-3'
rs2299007 Fwd		5'-TGTTGGTGAAGTGGAGAGAT-3'
rs2299007 Rev		5'-Biotin-ACAATAACAAACTTACATTCCTTCTAT-3'
rs2299007 Seq		5'AAGTGGAGAGATGGT-3'
rs17690965	C__11818506_20	Proprietary to ThermoFisher
HPRT	Mm03024075_m1	Proprietary to ThermoFisher
Kif3a	Mm00492876_m1	Proprietary to ThermoFisher
IL4	Mm00445259_m1	Proprietary to ThermoFisher
IL13	Mm00434204_m1	Proprietary to ThermoFisher
Irf4	Mm00516431_m1	Proprietary to ThermoFisher
Wnt7a	Mm00437356_m1	Proprietary to ThermoFisher
Kng2	Mm01343599_m1	Proprietary to ThermoFisher
Inhba	Mm00434339_m1	Proprietary to ThermoFisher
Ntkr2	Mm00435422_m1	Proprietary to ThermoFisher
Igfals	Mm01962637_s1	Proprietary to ThermoFisher
rs11740584 CpGA,B Fwd		5'-ATTTTAAATTATTTGGTTAGGTAAGGT-3'
rs11740584 CpGA,B Rev		5'-Biotin-CCAAACTAATCTCAAACTCTTAACC-3'
rs11740584 CpGA,B Seq		5'-GGTTAGGTAAGGTGG-3'
rs11740584 CpGC Fwd		5'-Biotin-AAATTTAGGTTTTTAATATAGATGTAAGT-3'

rs11740584 CpGC Rev		5’-ACCATTTACCTAATTAACTTTACATT-3’
rs11740584 CpGC Seq		5’-ACATTTTTTCTCCTATATTAATCC-3’
rs2299007 CpGA Fwd		5’-AAGGTTTAATGAAGAATTTTTAAAGATATA-3’
rs2299007 CpGA Rev		5’-Biotin-ACTTAAATCCTTAATTAACAATAACTCC-3’
rs2299007 CpGA Seq		5’-AGAATTTTTAAAGATATAAGTTTTT-3’
rs2299007 CpGB,C Fwd		5’-AGGAGTTATTGTTAATTAAGGATTTAAGTA-3’
rs2299007 CpGB,C Rev		5’-Biotin-TATTCAATTCAAAAAATTTTAATTCCTATT-3’
rs2299007 CpGB,C Seq		5’-AAAAGGGAAGGTATATATAG-3’
