# Supplementary material for: Disease-associated KIF3A variants alter gene methylation and expression impacting skin barrier and atopic dermatitis risk
Source: Nat Commun. 2020 Aug 14;11:4092. doi: 10.1038/s41467-020-17895-x (PMC7427989; doi:10.1038/s41467-020-17895-x)
Supplement: Supplementary file 3 — Reporting Summary [file 41467_2020_17895_MOESM3_ESM.pdf]

# nature research

Corresponding author(s): Gurjit K. Khurana Hershey

Last updated by author(s): Jul 20, 2020

# Reporting Summary

Nature Research wishes to improve the reproducibility of the work that we publish. This form provides structure for consistency and transparency in reporting. For further information on Nature Research policies, see our Editorial Policies and the Editorial Policy Checklist.

## Statistics

For all statistical analyses, confirm that the following items are present in the figure legend, table legend, main text, or Methods section.

| n/a | Confirmed | |
|---|---|---|
| ☐ | ☒ | The exact sample size ($n$) for each experimental group/condition, given as a discrete number and unit of measurement |
| ☐ | ☒ | A statement on whether measurements were taken from distinct samples or whether the same sample was measured repeatedly |
| ☐ | ☒ | The statistical test(s) used AND whether they are one- or two-sided<br>*Only common tests should be described solely by name; describe more complex techniques in the Methods section.* |
| ☐ | ☒ | A description of all covariates tested |
| ☐ | ☒ | A description of any assumptions or corrections, such as tests of normality and adjustment for multiple comparisons |
| ☐ | ☒ | A full description of the statistical parameters including central tendency (e.g. means) or other basic estimates (e.g. regression coefficient) AND variation (e.g. standard deviation) or associated estimates of uncertainty (e.g. confidence intervals) |
| ☐ | ☒ | For null hypothesis testing, the test statistic (e.g. $F$, $t$, $r$) with confidence intervals, effect sizes, degrees of freedom and $P$ value noted<br>*Give P values as exact values whenever suitable.* |
| ☒ | ☐ | For Bayesian analysis, information on the choice of priors and Markov chain Monte Carlo settings |
| ☒ | ☐ | For hierarchical and complex designs, identification of the appropriate level for tests and full reporting of outcomes |
| ☐ | ☒ | Estimates of effect sizes (e.g. Cohen's $d$, Pearson's $r$), indicating how they were calculated |

*Our web collection on statistics for biologists contains articles on many of the points above.*

## Software and code

Policy information about availability of computer code

| | |
|---|---|
| Data collection | No software was used for data collection. |
| Data analysis | Pyro Q-CpG (Version 1.0.11), Nikon Imaging Software Elements (Version 4.4) , Image J (Version 1.52). All analyses were performed in R software (Version 3.3.2). |

For manuscripts utilizing custom algorithms or software that are central to the research but not yet described in published literature, software must be made available to editors and reviewers. We strongly encourage code deposition in a community repository (e.g. GitHub). See the Nature Research guidelines for submitting code & software for further information.

## Data

Policy information about availability of data

All manuscripts must include a data availability statement. This statement should provide the following information, where applicable:

- Accession codes, unique identifiers, or web links for publicly available datasets
- A list of figures that have associated raw data
- A description of any restrictions on data availability

Tissue-specific KIF3A expression data for rs11740584 and rs2299007 were retrieved from the GTEx database, analysis release V7 (https://www.gtexportal.org). The whole-genome-sequencing data associated with this study are available through GEO accession GSE151706 [https://www.ncbi.nlm.nih.gov/geo/query/acc.cgi?acc=GSE151706]. The source data underlying Figs. 1c, 2a-d, 2f, 3a-c, 3e-f, 5a-b, and Supplementary Figures, 1b-c, 2b, 2e, 3a-b, d are provided as a Source data file.



April 2020

# Field-specific reporting

Please select the one below that is the best fit for your research. If you are not sure, read the appropriate sections before making your selection.

☒ Life sciences ☐ Behavioural & social sciences ☐ Ecological, evolutionary & environmental sciences

For a reference copy of the document with all sections, see nature.com/documents/nr-reporting-summary-flat.pdf

# Life sciences study design

All studies must disclose on these points even when the disclosure is negative.

| | |
|---|---|
| Sample size | We performed power calculations and choose a sample size to give us a power of at least 95%. |
| Data exclusions | We established the following exclusion criteria prior to analysis: when replicate values exceeded a coefficient of variation of 10, then the value that maximize the coeficient of variation was removed. We implemented the exclusion criteria on the allele specific PCR analysis (fIgure 2 panel F) where 2 replicate values from 2 different individuals were removed. |
| Replication | We analyzed skin cells taken from human subjects and we have incorporated strict methods to insure the identity and validity of these cells. All experiments were done 1-4 times as noted in the figure legends and each point was run in technical triplicates. All attempts of replication were successful except for Supplementary figure 3 panels a and b, where we saw variation between the first 3 repeats and therefore performed a 4 repeat to strength the findings. |
| Randomization | This is an observational study, randomization is not applicable. |
| Blinding | This is an observational study, blinding is not applicable. |

# Reporting for specific materials, systems and methods

We require information from authors about some types of materials, experimental systems and methods used in many studies. Here, indicate whether each material, system or method listed is relevant to your study. If you are not sure if a list item applies to your research, read the appropriate section before selecting a response.

## Materials & experimental systems

| n/a | Involved in the study | |
|---|---|---|
| ☐ | ☒ | Antibodies |
| ☒ | ☐ | Eukaryotic cell lines |
| ☒ | ☐ | Palaeontology and archaeology |
| ☐ | ☒ | Animals and other organisms |
| ☐ | ☒ | Human research participants |
| ☒ | ☐ | Clinical data |
| ☒ | ☐ | Dual use research of concern |

## Methods

| n/a | Involved in the study | |
|---|---|---|
| ☒ | ☐ | ChIP-seq |
| ☒ | ☐ | Flow cytometry |
| ☒ | ☐ | MRI-based neuroimaging |

## Antibodies

| | |
|---|---|
| Antibodies used | Keratin 5 (905501, Rabbit polyclonal, Biolegend), E-cadherin (180223, Mouse monoclonal IgG1kappa, Life technologies), Claudin 1 (37-4900, Mouse IgG1, Thermofisher Scientific), Ki67 (ab15580, Rabbit polyclonal, Abcam), CD3 (100303,Biolegend), Alexafluor 488 anti-rabbit (A-11008, Thermofisher Scientific), Alexafluor 488 (A-11029, Thermofisher Scientific), Alexafluor 594 anti-mouse (A-11032, Thermofhisher scientific). |
| Validation | All purchased antibodies have been described as suitable and independently validated for immunohistochemistry by the manufacturers. Keratin 5 polyclonal antibody Poly19055 is used for the in vitro examination of human biological samples using immunohistochemistry (IHC) methods for the qualitative identification of Keratin 5. E-cadherin monoclonal antibody reacts with human epithelial cadherin (E-cadherin, uvomorulin, Cell-CAM 120/80), a 120 kDa transmembrane glycoprotein. Claudin 1 antibody is specific for the C-terminal region of the claudin-1 protein. Ki67 Ab15580 antibody is batch tested in ICC/IHC. CD3 antibody is quality control tested by immunofluorescent staining with flow cytometric analysis. To validate specificity of the antibodies for our immunostaining experiments, we ran tissue section samples with no primary but only the appropriate secondary antibody in parallel with primary antibody containing samples in all of our experiments. |

## Animals and other organisms

Policy information about studies involving animals; ARRIVE guidelines recommended for reporting animal research

| | |
|---|---|
| Laboratory animals | Mouse, F1 of C57Bl/6 and 129 strains, females and males, 6-10 weeks of age. Kif3aK14Δ/Δ mice were maintained on an alternating |

| | |
|---|---|
| Laboratory animals | 12-hour light cycle with food and water ad libitum, the temperature was kept between 70-74F, humidity between 30-70%, and otherwise maintained and handled according to the Guide for the Care and Use of Laboratory Animals (Institute of Laboratory Animal Resources, National Research Council). |
| Wild animals | The study did not involve wild animals. |
| Field-collected samples | The study did not involve samples collected from the field. |
| Ethics oversight | All animal procedures were approved by the Institutional Animal Care and Use Committee (Cincinnati Children's Hospital Medical Center). |

Note that full information on the approval of the study protocol must also be provided in the manuscript.

## Human research participants

Policy information about studies involving human research participants

| | |
|---|---|
| Population characteristics | The GCPCR-KIF3A cohort includes a total of 56 participants (45 children aged 7 to 17 years old and 11 adults aged 18 to 26 years old) being 25% black and 66% male, from the Greater Cincinnati, Ohio metro area. |
| Recruitment | We recruited individuals based on genotype for KIF3A SNPs rs11740584 and/or rs2299007. We minimized self-selection bias by inviting all potential subjects with the genotypes of interest to participate and not disclosing to the participants the genetic results as they are not meaningful on an individual level. Since individuals with Atopic Dermatitis could potentially be more likely to participate, we matched the disease status on both genotype groups. |
| Ethics oversight | The study was approved by the Cincinnati Children's Hospital Medical Center Institutional Review Board under protocol number 2010-0438. |

Note that full information on the approval of the study protocol must also be provided in the manuscript.
