# Supplementary material for: Disease-associated KIF3A variants alter gene methylation and expression impacting skin barrier and atopic dermatitis risk
Source: Nat Commun. 2020 Aug 14;11:4092. doi: 10.1038/s41467-020-17895-x (PMC7427989; doi:10.1038/s41467-020-17895-x)
Supplement: Supplementary file 2 — Peer Review File [file 41467_2020_17895_MOESM2_ESM.docx]

Peer Review Information

**Journal:** Nature Communications

**Manuscript title:** Disease-associated KIF3A variants alter gene methylation and expression impacting skin barrier and atopic dermatitis risk

**Corresponding author name(s):** Dr Gurjit Khurana

**Editorial notes:**

**none**

**Reviewer comments & decisions:**

**Reviewer comments, first version:**

Reviewers' comments:

Reviewer #1 (Remarks to the Author):

The manuscript by Stevens et al. examine patients who are heterozygous or homozygous for the presence of either of two KIF3A SNP alleles that confer risk for developing asthma and AD. These alleles create new CpG sites that the authors predict may reduce expression of KIF3A as a result of hypermethylation. They examined DNA methylation in skin from adhesive tape strips and in nasal epithelial cells from these patients. They found that individuals with 1 copy of the alternate allele had an average of 52 and 43% methylation, and individuals with 2 copies of the alternate allele had a range of 25-78% methylation at the newly created CpG sites. They used allelic-specific qPCR to determine that increased methylation was correlated with decreased expression of KIF3A compared to the reference allele. They also showed that methylation levels of the SNP sites were associated with higher levels of transepidermal water loss (TEWL).
To gain mechanistic insight into how decreased levels of expression of KIF3A impacts epidermal barrier function, they used a mouse model with an epidermal-specific deletion of KIF3A (Kif3aK14Δ/Δ). As observed in patients harboring either of the SNP alleles, mice deficient in expression of KIF3A in the skin exhibited high TWEL levels compared to control mice. The epidermis of Kif3aK14Δ/Δ mice was markedly thicker than controls and hyperplastic as confirmed by Ki67 staining. Further analysis of the epidermis of Kif3aK14Δ/Δ mice revealed expansion of the basal layer as determined by K5 staining and altered localization of adherens and tight junction proteins visualized by staining for E-cadherin and claudin-1.
Kif3aK14Δ/Δ mice did not develop a spontaneous AD phenotype. However, following exposure to the cutaneous allergen (Aspergillus fumigatus), these mice developed increased TWEL, and an increase in epidermal thickness.
Overall, the data are very convincing and the Kif3aK14Δ/Δ mouse model seems to phenocopy well patients that express the risk alleles that result in reduced expression of KIF3A.
I only have a few comments:
1. There are issues with the labels for panels in Fig. 3d and Fig. 5c.
2. The images of H&E staining in Fig. 5c should be rotated placing the epidermis on top.
3. Were the authors able to show the presence of Aspergillus fumigatu-specific IgE in the serum of Kif3aK14Δ/Δ mice exposed to this cutaneous allergen?


Reviewer #2 (Remarks to the Author):

The manuscript by Stevens et al describes results of a functional study of KIF3A genetic variants in atopic dermatitis (AD). Previous work showed that genetic variation in the cilia structural gene KIF3A was associated with AD, asthma, and the atopic march by many studies and lung-specific deletion of Kif3a resulted in barrier dysfunction and a Th2 allergen response. The authors noted that among the KIF3A SNPs associated with asthma and AD, a third of them create novel CpG sites that can undergo methylation. In this manuscript, they show that:
1. the alternate alleles of rs11740584 and rs2299007 (both intronic) had 14-22% decreased KIF3A expression in GTEx data.
2. DNA methylation in skin from adhesive tape strips and in nasal epithelial cells from human subjects showed that individuals with 2 reference alleles for rs11740584 or rs2299007 had low or negligible methylation while individuals with one or two copies of the alternate allele had methylation (wide range 16-80%) at the newly-created CpG sites.
3. rs17690965 located within a KIF3A exon, in strong LD with rs11740584 (r2=0.96), associated with a 16% decrease in KIF3A expression for the alternate allele G relative to the reference allele C in two heterozygote individuals using allele-specific qPCR. No coding genetic variant in strong LD was identified for rs2299007 so this assay was not performed.
4. Methylation levels in the skin of the novel CpG sites created by rs11740584 and rs2299007 were significantly associated with skin barrier dysfunction as assessed by TEWL values.
5. Mice with epidermis-specific deletion of Kif3a had higher TEWL, overproliferation of the basal cell layer. disrupted junctional proteins, and increased susceptibility to develop AD in the cutaneous A. fumigatus model.

Overall, the manuscript is well written and data are presented in a logical and easy to follow fashion. The results are interesting and shed light on the role of KIF3A in AD. Data on the functional relevance of KIF3A in AD (cell and animal models) are very strong. I, however, struggle greatly with the interpretation and significance of the methylation data:
1. Why is it surprising that CpGs are methylated upon creation when they are in introns and gene bodies are generally methylated in the human genome?
2. How can comparison of no methylation at a non-CpG site (reference allele) to methylation at a newly created CpG site (alternate allele) be valid? It is completely confounded by the presence of the variant so I am not sure that is a statistically valid test to perform (Figure 2). Would it be tested as an interaction of methylation level and presence of the variant? I am not sure how to best test this but I don’t think a simple Wilcoxon test can be used.
3. Are other CpGs in these introns similarly methylated?
4. Are there CpGs in enhancers, TF binding sites, or other functional areas of the genome/epigenome?


Reviewer #3 (Remarks to the Author):

Understanding the functional consequence of genetic risk in atopic dermatitis is important for understanding disease mechanisms and identifying therapeutic strategies. The authors have identified a novel and potentially important role of KIF3A in skin barrier homeostasis and have provided evidence of SNP dependent methylation changes associated with intronic KIF3A variants.

The data is clearly presented is of good quality, however, a number of additional experiments/analyses are required to fully support the authors conclusions.
[1] Allelic PCR should be performed in keratinocytes in addition to NE cells and in at least 3 subjects. In addition the expression of KIF3A and proximal cytokine cluster genes should be compared between the 3 genotypes represented in the GCRCR-KIF3A cohort.

[2] A much more detailed characterisation of the KIF3A knockout mouse is required to determine the molecular basis of skin barrier disruption. Suggested unbiased analyses include RNAseq or global proteomic analysis. Moreover detailed characterisation of the skin ultrastructure would be of benefit.

[3] The authors should justify their use of aspergillus fumigatus in the mouse model because this is not a classically associated allergen in AD.

In addition the text embedded in most of the figures requires re-formatting because it has scrambled.

**Author rebuttal, first version:**

**COMMENTS FROM REVIEWER #1:**

The manuscript by Stevens et al. examine patients who are heterozygous or homozygous for the presence of either of two KIF3A SNP alleles that confer risk for developing asthma and AD. These alleles create new CpG sites that the authors predict may reduce expression of KIF3A as a result of hypermethylation. They examined DNA methylation in skin from adhesive tape strips and in nasal epithelial cells from these patients. They found that individuals with 1 copy of the alternate allele had an average of 52 and 43% methylation, and individuals with 2 copies of the alternate allele had a range of 25-78% methylation at the newly created CpG sites. They used allelic-specific qPCR to determine that increased methylation was correlated with decreased expression of KIF3A compared to the reference allele. They also showed that methylation levels of the SNP sites were associated with higher levels of transepidermal water loss (TEWL).

To gain mechanistic insight into how decreased levels of expression of KIF3A impacts epidermal barrier function, they used a mouse model with an epidermal-specific deletion of KIF3A (Kif3aK14Δ/Δ). As observed in patients harboring either of the SNP alleles, mice deficient in expression of KIF3A in the skin exhibited high TWEL levels compared to control mice. The epidermis of Kif3aK14Δ/Δ mice was markedly thicker than controls and hyperplastic as confirmed by Ki67 staining. Further analysis of the epidermis of Kif3aK14Δ/Δ mice revealed expansion of the basal layer as determined by K5 staining and altered localization of adherens and tight junction proteins visualized by staining for E-cadherin and claudin-1. Kif3aK14Δ/Δ mice did not develop a spontaneous AD phenotype. However, following exposure to the cutaneous allergen (Aspergillus fumigatus), these mice developed increased TWEL, and an increase in epidermal thickness.

Overall, the data are very convincing and the Kif3aK14Δ/Δ mouse model seems to phenocopy well patients that express the risk alleles that result in reduced expression of KIF3A.

**Reply: We thank the Reviewer for the kind comments.**

I only have a few comments:

1. There are issues with the labels for panels in Fig. 3d and Fig. 5c.

**Reply: We had a problem with the Mac to PC conversion, the text is now fixed on all figures.**

2. The images of H&E staining in Fig. 5c should be rotated placing the epidermis on top. **Reply: The images have been rotated and now the epidermis is above the dermis.**
3. Were the authors able to show the presence of Aspergillus fumigatus-specific IgE in the serum of Kif3aK14Δ/Δ mice exposed to this cutaneous allergen?

**Reply: We checked specific IgE levels after 1 week of patching, and although the timing is sufficient to induce water loss and epidermal thickness, it is not enough for an adaptative immune response, so we did not see statistically significant differences on specific IgE compared to saline patched animals. When we check the mice 2 weeks later, we are able to detect *Aspergillus fumigatus*-specific IgE in the Asp-patched animals compared to the saline-patched animals.**

**COMMENTS FROM REVIEWER #2:**

The manuscript by Stevens et al describes results of a functional study of KIF3A genetic variants in atopic dermatitis (AD). Previous work showed that genetic variation in the cilia structural gene KIF3A was associated with AD, asthma, and the atopic march by many studies and lung-specific deletion of Kif3a resulted in barrier dysfunction and a Th2 allergen response. The authors noted that among the KIF3A SNPs associated with asthma and AD, a third of them create novel CpG sites that can undergo methylation. In this manuscript, they show that:

1. the alternate alleles of rs11740584 and rs2299007 (both intronic) had 14-22% decreased KIF3A expression in GTEx data.
2. DNA methylation in skin from adhesive tape strips and in nasal epithelial cells from human subjects showed that individuals with 2 reference alleles for rs11740584 or rs2299007 had low or negligible methylation while individuals with one or two copies of the alternate allele had methylation (wide range 16-80%) at the newly-created CpG sites.
3. rs17690965 located within a KIF3A exon, in strong LD with rs11740584 (r2=0.96), associated with a 16% decrease in KIF3A expression for the alternate allele G relative to the reference allele C in two heterozygote individuals using allele-specific qPCR. No coding genetic variant in strong LD was identified for rs2299007 so this assay was not performed.
4. Methylation levels in the skin of the novel CpG sites created by rs11740584 and rs2299007 were significantly associated with skin barrier dysfunction as assessed by TEWL values.
5. Mice with epidermis-specific deletion of Kif3a had higher TEWL, overproliferation of the basal cell layer. disrupted junctional proteins, and increased susceptibility to develop AD in the cutaneous A. fumigatus model.

Overall, the manuscript is well written and data are presented in a logical and easy to follow fashion. The results are interesting and shed light on the role of KIF3A in AD. Data on the functional relevance of KIF3A in AD (cell and animal models) are very strong. I, however, struggle greatly with the interpretation and significance of the methylation data:

**Reply: We thank the Reviewer for the kind comments. We have addressed the Reviewer’s comments regarding the methylation data (see below).**

1. Why is it surprising that CpGs are methylated upon creation when they are in introns and gene bodies are generally methylated in the human genome?

**Reply: The reviewer is correct that CpGs are generally methylated in the human genome. In fact, in the human genome, where ~28million CpGs are found, 60-80% of them are methylated (Smith and Meissner, Nature, 2013). However, this means that 20-40% are not methylated, thus, although methylation occurs on CpG sites most of the time, not all CpG sites are methylated. We needed to demonstrate this explicitly and our data demonstrate that this newly generated site is methylated *in vivo* in relevant cells. Further, the level of methylation varied among individuals. This is an important point that the Reviewer may not be appreciating. It is not as simple as if the allele is present, it will be methylated. There is some additional regulation taking place and we go on to show that among those who carry the risk allele, their TEWL correlated with the methylation level.**

2. How can comparison of no methylation at a non-CpG site (reference allele) to methylation at a newly created CpG site (alternate allele) be valid? It is completely confounded by the presence of the variant so I am not sure that is a statistically valid test to perform (Figure 2). Would it be tested as an interaction of methylation level and presence of the variant? I am not sure how to best test this but I don’t think a simple Wilcoxon test can be used.

**Reply: We understand the Reviewer’s confusion as the site does not exist in individual with the reference allele and thus cannot be methylated. There may be some confusion regarding this as Wilcoxon rank sum was not used for this analysis. We have discussed this further with our senior statistician (Dr. Lisa J Martin) and she confirmed that the one-way ANOVA statistical approach employed to investigate association between methylation and genotype is appropriate and valid to test whether methylation is associated with genotype. Wilcoxon rank sum was previously employed to test allele expression of *Kif3a* in nasal epithelial cells. This is now revised by Dr. Lisa Martin and we have implemented a linear mixed model for both nasal epithelial cells and primary keratinocytes in Figure 2F. Our presented data are highly important because the presence of a CpG site does not automatically mean that it is methylated; our data clearly demonstrate that methylation is strongly associated with genotype. Further, the allele specific PCR analysis demonstrates differential allelic expression supporting a potential role of methylation on gene expression. Further, we provide evidence that methylation at this site is clinically relevant because variation in the level of DNA methylation at this CpG correlates with TEWL in human subjects.**

3. Are other CpGs in these introns similarly methylated?

**Reply: We thank the Reviewer for this comment. It is a very important point as it was possible that our observations were due, in part, to impact of our risk SNP alleles on methylation at neighboring CpG sites. To address this, we have conducted additional experiments in order to quantify methylation levels at 6 CpG sites neighboring rs11740584 or rs2299007. This new data has been added to the revised manuscript in the supplementary figure 1. There were no differences in methylation levels across the genotype groups at any of the additional CpG sites tested in both nasal epithelial and skin cells. Thus, only the CpG sites directly created by the SNPs demonstrate altered methylation by genotype and the genotype does not impact neighboring SNPs. These data further support our conclusions.**

4. Are there CpGs in enhancers, TF binding sites, or other functional areas of the genome/epigenome? **Reply: We thank the Reviewer for this helpful comment. In order to address this further, we have enlisted a new collaborator, Dr. Matt Weirauch, a computational biologist and expert in transcription factor and epigenomic analyses. He is now included as a co-author and together we have conducted *in silico* analyses to directly address this Reviewer comment. Our findings, included in the supplementary Table 1, reveal that the rs2299007 non-reference (risk) G allele creates a perfect MYC binding site. Specifically, the rs2299007 non-reference (risk) G allele creates a CACGTG E-box (ie in the presence of the risk allele). Basic helix-loop-helix (bHLH) family TFs such as MYC can bind the CACGTG E-box when**

**the CpG is methylated (Yin et al, 2017; PMID: 28473536). So, the prediction would be that the rs2299007 non-reference risk allele creates a new binding site for MYC, and MYC binds this site in keratinocytes. Further, rs2299007 is located inside of a MYC ChIP-seq peak in BJ cells (foreskin fibroblast cell line) (Cell 2012 Nov 21;151(5):994-1004. PMID: 23159369). Collectively, these findings add a novel layer to what is known currently regarding *KIF3A* transcriptional regulation and further highlight the relevance our findings. Neither SNP has histone marks in relevant (skin or lung) cell types. The non-reference (risk) allele for rs11740584 creates a binding site for the transcription factor ZSCAN26 so we predict that ZSCAN26 would bind more strongly in the presence of the risk allele. We have added this new data to the revised manuscript and are currently investigating the transcriptional regulation of *KIF3A* in the context of these SNPs as part of a follow up study.**

**COMMENTS FROM REVIEWER #3:**

Understanding the functional consequence of genetic risk in atopic dermatitis is important for understanding disease mechanisms and identifying therapeutic strategies. The authors have identified a novel and potentially important role of KIF3A in skin barrier homeostasis and have provided evidence of SNP dependent methylation changes associated with intronic KIF3A variants.

The data is clearly presented is of good quality, however, a number of additional experiments/analyses

are required to fully support the authors conclusions.

**Reply: We thank the Reviewer for the kind comments.**

[1] Allelic PCR should be performed in keratinocytes in addition to NE cells and in at least 3 subjects. In addition the expression of KIF3A and proximal cytokine cluster genes should be compared between the 3 genotypes represented in the GCRCR-KIF3A cohort.

**Reply: We thank the Reviewer for this important comment. We have added a third subject to the NE cell allelic PCR, and the data is included in the revised manuscript in Figure 2, Panel F. With the new subject, the average expression decreased from 16% to 17%. In order to address the Reviewer’s suggestion that we also perform allelic PCR in keratinocytes, we enlisted Dr. Dorothy Supp, an investigator with over 20 years of experience in human primary keratinocyte biology. From her, we were able to identify primary keratinocyte derived from 3 donors who are heterozygous for rs11740584. We performed allele-specific PCR and this data is now included in the revised manuscript (Figure 2, Panel F). The data show that the presence of rs11740584 risk allele results in an average 19% decrease in *KIF3A* expression. These data agree nicely with the data we had previously obtained in NE cells. As the Reviewer points out, the KIF3A gene falls in the cytokine cluster. We have not seen any impact of *KIF3A* genotypes on *IL4* expression in human NE cells. We do see slightly decreased IL-4 expression in the *Kif3a^K14∆/∆^* mice compared to the *Kif3a^+/+^* mice as shown in Supplementary Figure 3, Panel A, however this does not explain the mouse phenotype that we observe, and this difference was not observed in the new RNAseq analysis of the *Kif3a^K14∆/∆^* mice.**

[2] A much more detailed characterisation of the KIF3A knockout mouse is required to determine the molecular basis of skin barrier disruption. Suggested unbiased analyses include RNAseq or global proteomic analysis. Moreover detailed characterisation of the skin ultrastructure would be of benefit. **Reply: As suggested by the Reviewer, we have conducted an unbiased global RNAseq analysis and this data is now added to the revised manuscript in Supplemental Figure 2. In the absence of *Kif3a*, the top functional terms for the downregulated genes are defense mechanisms and cell adhesion, while the top functional terms for the upregulated genes are growth factor binding and basolateral plasma membrane. The RNAseq data indicates morphological changes which supports our immunostaining**

**data whereby expression of adhesion markers E-Cadherin and Claudin-1 are decreased, while expression of basal markers including Keratin-5 is increased. We have also re-reviewed the H&E staining with our pathologist and collaborator Dr. Kathryn Wikenheiser Brokamp, and she confirmed that there is no evidence of inflammation in the skin of these mice. The RNAseq data confirm this as we do not see evidence of increased inflammation. This new data has now been included. Given the Covid-19 situation, we have not been able to perform ultrastructural studies, but we hope that the significant additional studies which we have conducted will satisfy the Reviewer.**

[3] The authors should justify their use of aspergillus fumigatus in the mouse model because this is not a classically associated allergen in AD.

**Reply: This AD model is well documented and used in the literature (Akei et al., 2005; Akei et al., 2006; Brandt et al., 2013). We chose to use this model because when compared to other models, this one best modeled AD and it is well established in our laboratory. In this model, cutaneous applications of *Asp. fumigatus* result in increased transepidermal water loss, allergic sensitization, skin erythema, skin thickness, and Th2 responses, all landmarks of atopic dermatitis, without the need for prior mechanical tape stripping or skin perturbation with shaving creams (Akei et al., 2005; Akei et al., 2006; Brandt et al., 2013). The requirement for prior skin perturbations in the other models render them less optimal. This is now clarified in the text.**

In addition the text embedded in most of the figures requires re-formatting because it has scrambled. **Reply: This has been fixed in all of the figures.**

**Reviewer comments, second version:**

REVIEWERS' COMMENTS:

Reviewer #2 (Remarks to the Author):

The authors have done a very nice job addressing all concerns raised in the review process. They have added additional data, analyses (and collaborators to perform them), and have clarified some of the points. I don't have any additional feedback, this is a nice manuscript.


Reviewer #3 (Remarks to the Author):


The authors have addressed my prior comments to a high standard.

There are minor formatting issues with the text in figures S2 and S3 which should be corrected.

Dr. Martina Elias

**Author rebuttal, second version:**

REVIEWERS' COMMENTS:

Reviewer #2 (Remarks to the Author):

The authors have done a very nice job addressing all concerns raised in the review process. They have added additional data, analyses (and collaborators to perform them), and have clarified some of the points. I don't have any additional feedback, this is a nice manuscript.
We thank the Reviewer for the kind comments.

Reviewer #3 (Remarks to the Author):


The authors have addressed my prior comments to a high standard.

We thank the Reviewer for the kind comments.

There are minor formatting issues with the text in figures S2 and S3 which should be corrected.

Done.

**Reviewer comments, third version:**

**Author rebuttal, third version:**
